# Remodeling the Physicochemical and Pharmacokinetic Properties of PROTAC via Lipid Nanodisks for Cancer Therapy

**DOI:** 10.1002/advs.202501384

**Published:** 2025-06-29

**Authors:** Meichen Pan, Chunrong Yang, Zhongliang Fu, Yuchen Yang, Ying Zhuo, Hongwei Hou, Jinghong Li

**Affiliations:** ^1^ School of Biomedical Sciences Hunan University Changsha Hunan 410082 China; ^2^ Department of Chemistry Center for BioAnalytical Chemistry Key Laboratory of Bioorganic Phosphorus Chemistry & Chemical Biology Tsinghua University Beijing 100084 China; ^3^ Key Laboratory of Luminescence Analysis and Molecular Sensing (Southwest University) Ministry of Education College of Chemistry and Chemical Engineering Southwest University Chongqing 400715 China; ^4^ Beijing Life Science Academy Beijing 102209 China; ^5^ New Cornerstone Science Laboratory Shenzhen 518054 China; ^6^ Center for BioAnalytical Chemistry Hefei National Laboratory of Physical Science at Microscale University of Science and Technology of China Hefei 230026 China

**Keywords:** cancer therapy, lipid nanodisk, nanodelivery, prodrug, PROTAC

## Abstract

Proteolysis targeting chimera (PROTAC), as an emerging approach for target protein degradation based on the intracellular ubiquitin‐protease system, is characterized by catalytic and reusability over traditional inhibitors. However, PROTACs are fraught with pharmacokinetic dangers due to poor water solubility and membrane permeability, further posing a huge challenge to the clinical potential. Herein, a nanodelivery system is developed that is elaborately decorated with prodrugs to improve the physicochemical properties and therapeutic efficacy of PROTAC. The nanodelivery, lipid nanodisk (LND), is readily assembled from three commercially available phospholipids, and shows high stability and long circulation time in vivo. The lipid‐derived prodrug realizes prolonged retention and precise release of PROTACs at tumor sites under the endogenous stimulus. Collectively, the LND loaded with MZ1 prodrug (LND‐MZ1) presents enhanced biocompatibility, improves intracellular accumulation, and superior tumor penetration capacity, enabling more potent and targeted PROTAC therapy with enhanced specificity in vivo. LND‐MZ1 shows a more significant anti‐tumor effect in the xenograft tumor model, even at a one‐tenth dose of the parent PROTACs under the same therapeutic schedule. Overall, the LND‐based nanomedicine paves the way for remodeling the physicochemical and pharmacokinetic properties of various drugs to expand the therapeutic scope.

## Graphical abstract

To solve the challenge of PROTACs in clinical potential due to poor water solubility and membrane permeability, a nanodelivery system based on lipid nanodisks loaded with MZ1 prodrug (LND‐MZ1) to remodel the physicochemical and pharmacokinetic properties of PROTAC MZ1 is elaborately proposed.

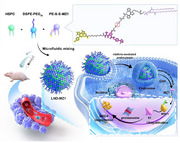

## 1 Introduction

Proteolysis targeting chimera (PROTAC) as an emerging tool for target protein degradation, is characterized by catalytic and reusability with the endogenous ubiquitin‐protease system.^[^ ^1^ ^]^ PROTAC chemically induces the proximity of a protein of interest (POI) and an E3 ligase, mediates the formation of ternary complexes, and leads to the ubiquitination of the POI, which is recognized and degraded by the intracellular 26S proteasome.^[^ ^1^, ^2^ ^]^ Different from small‐molecule inhibitors by the occupancy‐driven mode of action, PROTAC induces the degradation of target proteins through event‐driven modalities, allowing substoichiometric drug concentrations for activity and greatly expanding the range of target proteins.^[^ ^1^, ^3^ ^]^ In addition, PROTAC directly degrades the pathogenic target protein in a catalytic dose rather than merely inhibiting its function, which is a more thorough blockade method of protein function.^[^ ^4^ ^]^ This mechanism circumvents compensatory protein upregulation while significantly reducing the likelihood of drug resistance development. With the continuous discovery of E3 ligands, small molecule PROTACs were later developed to improve the permeability, oral bioavailability, and stability of the early PROTACs with peptide bonds.^[^ ^5^ ^]^ However, most PROTACs have a high molecular weight (> 800 Da) and a large polar surface area, which lacks adherence to Lipinski's Rule of Five (Ro5).^[^ ^6^ ^]^ Therefore, PROTACs have been considered as “pharmacokinetically dangerous” molecules with poor water solubility and membrane permeability, which further poses a huge challenge to the clinical efficacy of PROTACs.

Nanodelivery, which is a breakthrough means of remodeling the physicochemical properties of the drug, enables the modulation of biodistribution and enhances tumor accumulation, thereby reducing off‐target toxicity.^[^ ^7^ ^]^ Various carriers such as polymer nanoparticles,^[^ ^8^ ^]^ inorganic nanoparticles,^[^ ^9^ ^]^ and lipid‐based nanomaterials^[^ ^10^ ^]^ have been utilized as nanodelivery systems to enhance the stability and solubility of PROTAC, promote transport across biological barriers, extend cycle time, and achieve better efficacy and safety.^[^ ^11^ ^]^ Among these, lipid‐based nanomaterials with structures similar to cell membranes and remarkable biocompatibility, are fascinating candidates for constructing nanodelivery systems, with which both hydrophilic and hydrophobic drugs can be delivered to the tumor.^[^ ^12^ ^]^ However, the direct encapsulation of drugs is unstable in the package, and prone to systemic toxicity due to early leakage, resulting in only a small fraction of the drugs working at the tumor site. In addition, the prodrug strategies have also been proposed to enhance the therapy efficacy and safety of PROTAC.^[^ ^11^, ^13^ ^]^ The PROTAC prodrugs under the manipulation of endogenous tumor microenvironment or exogenous stimuli,^[^ ^14^ ^]^ such as X‐ray,^[^ ^15^ ^]^ light,^[^ ^16^ ^]^ and ultrasound,^[^ ^17^ ^]^ can readily release the activated PROTAC and reduce the off‐tissue toxicity. An excellent delivery system should steadily deliver drugs to tumor tissues and precisely release drugs in tumor cells to minimize adverse side effects, which can be achieved by ingeniously decorating the nanodelivery with PROTAC prodrugs through covalent modification.

**Scheme 1:**
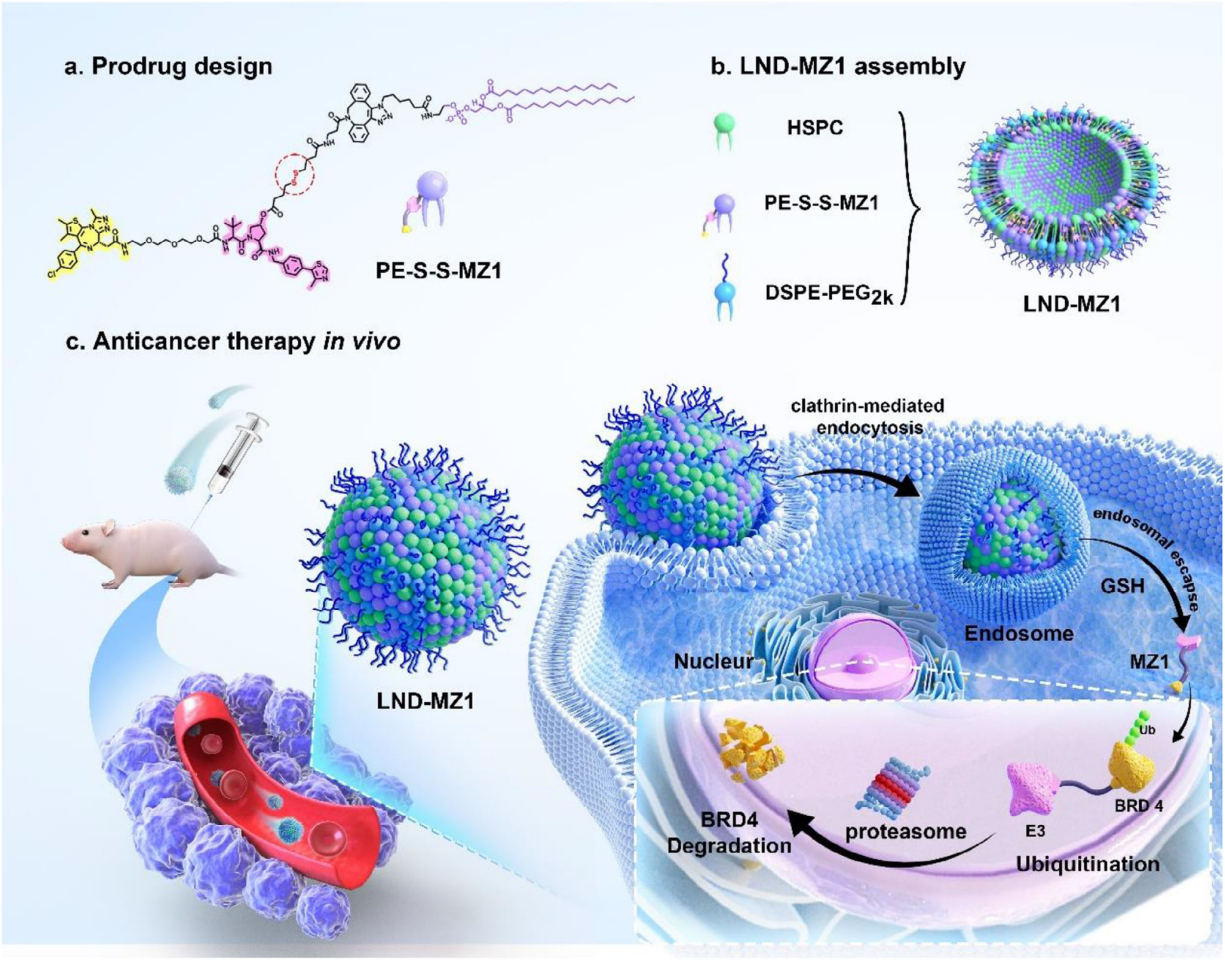
Schematic illustration of the anticancer mechanism of LND‐MZ1. a) Prodrug structure of PE‐S‐S‐MZ1 based on click chemistry. b) Formulation and assembly process of LND‐MZ1, containing HSPC, PE‐S‐S‐MZ1 and DSPE‐PEG_2K_. c) LND‐MZ1 nanodelivery for tumor‐specific BRD4 degradation and efficient antitumor therapy in vivo. LND‐MZ1 is systemically administered to mice and specifically enters tumor cells, facilitating increased PROTAC accumulation at the tumor site and prolonged systemic circulation time. The payload of LND‐MZ1, PE‐S‐S‐MZ1 as a prodrug, is site‐specifically activated to efficiently release MZ1 under intracellular GSH reduction conditions, resulting in rapid degradation of the target protein BRD4 by the endogenous proteasome and potent tumor growth inhibition at one‐tenth of the drug dose.

In this work, we developed a lipid nanodisk (LND) nanodelivery to compensate for the physicochemical deficiencies of PROTACs in druggability and further improve the bioavailability. As a proof of concept, PROTAC MZ1 was covalently attached to phosphatidyl ethanolamine (PE) lipid to synthesize PE‐S‐S‐MZ1. As a disulfide‐bridged prodrug, PE‐S‐S‐MZ1 not only blocked the protein degradation function of MZ1, but also modulated the physicochemical properties of MZ1 by improving hydrophilicity and biocompatibility. Moreover, under the reduction of overexpressed glutathione (GSH) in tumor cells,^[^ ^18^ ^]^ the disulfide bond was broken and PE‐S‐S‐MZ1 precisely released MZ1. To load the prodrug on LND, PE‐S‐S‐MZ1 was identified with two other commercially available phospholipids to easily assemble LND loaded with PE‐S‐S‐MZ1 (LND‐MZ1) by flash mixing. The LND‐MZ1 was cellularly internalized with high efficiency and showed accelerated protein degradation in tumor cells. In vivo, the LND‐MZ1 exhibits excellent tumor penetration, prolonged pharmacokinetics and enhanced therapeutic efficacy, ultimately reducing off‐target toxicity of MZ1 (**Scheme** **1**). Collectively, the LND as a versatile nanodelivery system enables PROTAC to achieve more rapid and durable protein degradation at the tumor site, synergistically inhibiting the tumor growth at a reduced dose.

## 2 Results and Discussion

### 2.1 Design and Synthesis of PE‐S‐S‐MZ1

PROTAC is a bifunctional molecule consisting of two ligands, one binding to a POI and the other recruiting an E3 ubiquitin ligase, with a linker in the middle. Rational drug design and iterative optimization could obtain PROTAC with higher selectivity, better protein degradation and safety. However, the multi‐component nature of PROTAC poses challenges in solubility and druggability, which have inspired researchers to explore simple and effective prodrugs or delivery strategies to improve pharmacodynamics and bioavailability.

**Figure 1:**
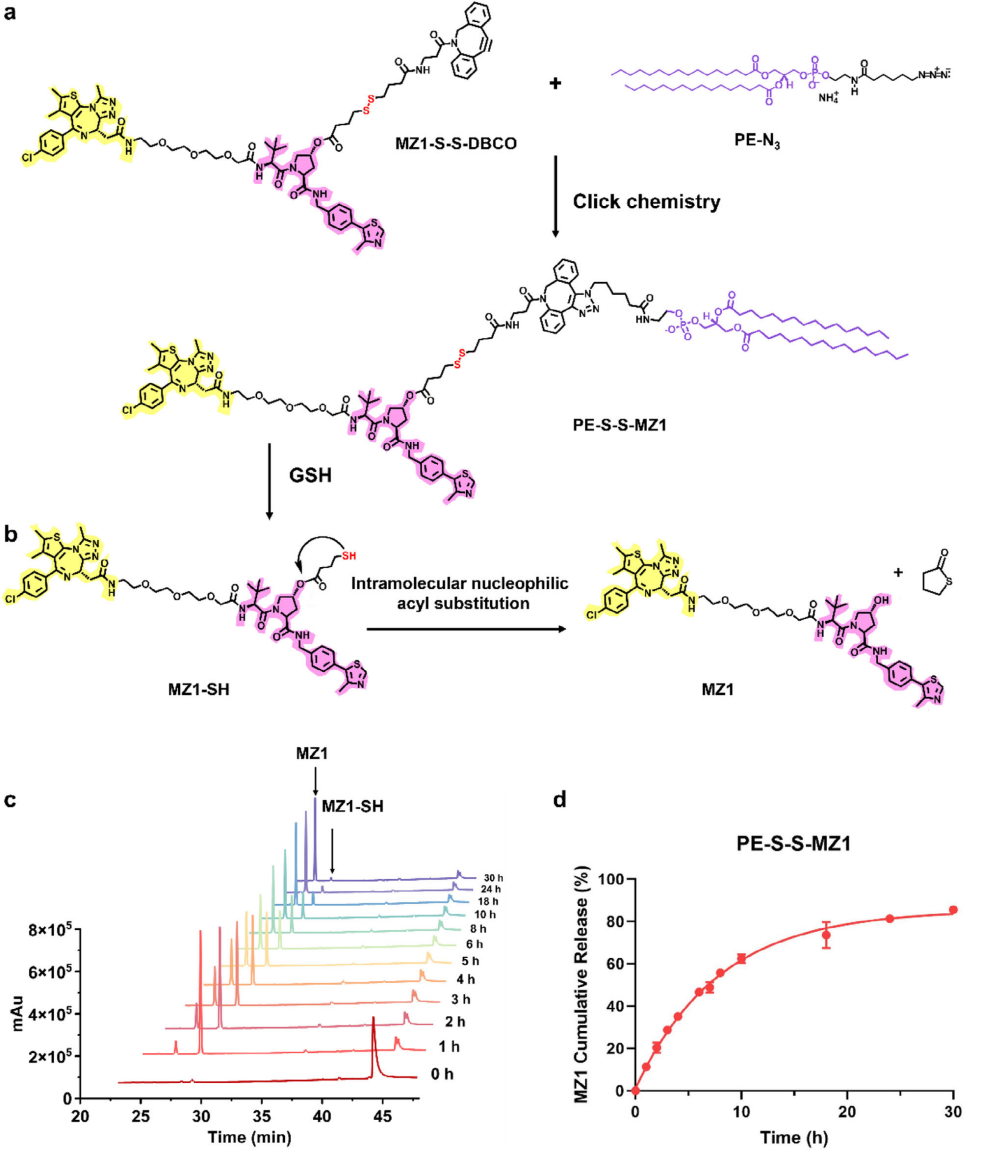
Synthesis and activation of PE‐S‐S‐MZ1. Schematic illustrations of a) the synthesis process of the prodrug PE‐S‐S‐MZ1 by the click chemistry of PE‐N_3_ and MZ1‐DBCO and b) the GSH‐triggered release mechanism of MZ1 by reduction and intramolecular nucleophilic acyl substitution. c) HPLC chromatograms and d) quantitative percentage curve of the released MZ1 from 100 µm PE‐S‐S‐MZ1 by incubation with 40 mm DTT for different times within 30 h at 37 °C. The detection wavelength was 254 nm. The data are presented as mean ± s.d. *n* = 3.

To enable spatiotemporal activation of PROTACs within tumor cells, a disulfide‐linked MZ1 prodrug (PE‐S‐S‐MZ1) was elaborately designed. Bromodomain and extraterminal domain (BET) family proteins, including BRD2, BRD3, BRD4 and BRDT, specifically read highly conserved acetyl‐lysine in histones.^[^ ^19^ ^]^ These proteins have become promising targets for inhibiting the expression of essential oncogenes. MZ1, a typical PROTAC for BRD4, conjugates a BET inhibitor JQ1 to a VHL ligand VH032 via a three‐unit PEG linker, which has been used as a template for modification to construct effective PROTAC delivery. According to the crystal structure of the Brd4^BD2^–MZ1–VHL complex, the hydroxyl group of VH032 plays a crucial role in the recruitment of the E3 ligase.^[^ ^19^, ^20^ ^]^ Therefore, the hydroxyl site provides the opportunity for prodrug design by blocking the hydroxyl group.^[^ ^18b^ ^]^ Given the abundant GSH as an endogenous biomarker in tumor cells,^[^ ^21^ ^]^ the disulfide bond was employed as a cleavable linkage in response to the reduced microenvironment to activate the PROTAC.^[^ ^22^ ^]^ The detailed synthesis process of PE‐S‐S‐MZ1 is outlined in Scheme S1 (Supporting Information). Specifically, the MZ1 was blocked by 4,4′‐dithiodibutyric acid via an esterification reaction and disulfide linkage was also introduced at the same time to produce MZ1‐S‐S‐COOH. Afterwards, the exposed carboxyl was further conjugated to a diphenylcycloctene (DBCO)‐based molecule with an amino group attached by an amide condensation reaction. Then, the click chemistry reaction was triggered between the MZ1‐conjugating DBCO group (MZ1‐S‐S‐DBCO) and PE lipid‐conjugating azide group (PE‐N_3_) to form the final PE‐S‐S‐MZ1 molecule (**Figure** **1**a). The structures of these synthetic compounds were confirmed by ^1^H NMR spectroscopy and high‐resolution mass spectrometry (HRMS) (Figures S1–S5, Supporting Information).

The schematic of the GSH‐triggered release of MZ1 is illustrated in Figure 1b. As reported, under the reduction role of GSH, the disulfide linkage is broken to produce a reaction intermediate containing the sulfhydryl group and further intramolecular nucleophilic acyl substitution occurred to release the drug.^[^ ^23^ ^]^ To confirm this, the ultraperformance liquid chromatography‐mass spectrum (UPLC‐MS) was applied to evaluate the release process of MZ1. Taking consideration of the stability and reduction, DL‐dithiothreitol (DTT), a frequently used analogue of GSH, was used to treat PE‐S‐S‐MZ1 for 2 h. In addition to the peak of MZ1 (*R*_T_ = 15.07 min), a new peak (*R*_T_ = 17.24 min) appeared and the corresponding MS peak was 1104.6 (Figure S6, Supporting Information), which proved the production of intermediates (MZ1‐SH) primarily, consistent with the previous reports.^[^ ^24^ ^]^ With the extension of reaction time, MZ1‐SH released a five‐member ring of thiolactone to produce active MZ1 (Figure 1c). As shown in Figure 1d, the release ratio was up to 85.6% at 30 h according to the standard curves of MZ1 (Figure S7, Supporting Information) and PE‐S‐S‐MZ1 (Figure S8, Supporting Information).

### 2.2 Assembly of LND‐MZ1 and In Vitro Release of MZ1

**Figure 2:**
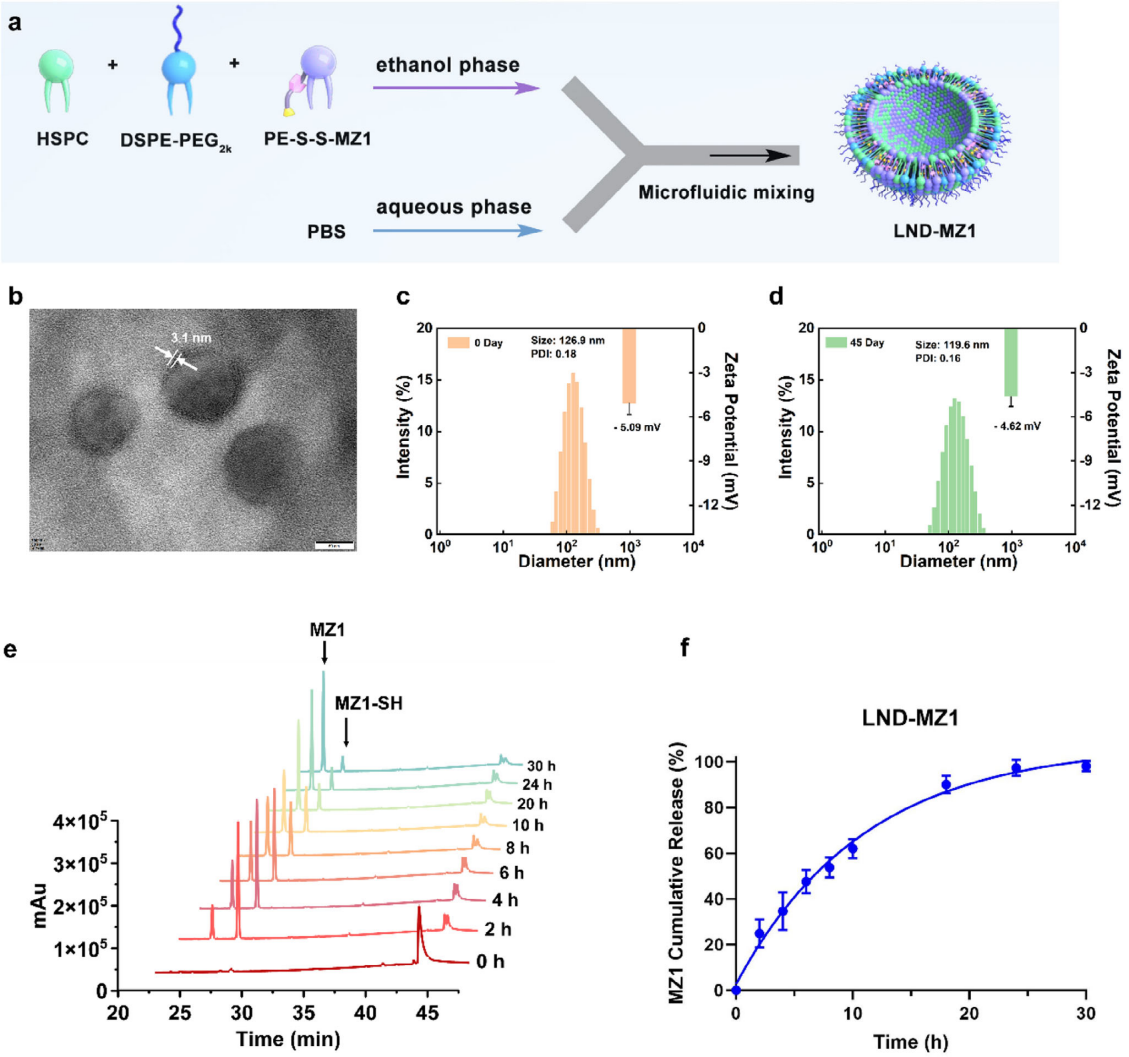
Assembly process, characterization and MZ1 release of LND‐MZ1. a) Schematic illustration of the LND‐MZ1 formulation by a microfluidic device. The ethanol phase containing HSPC, PE‐S‐S‐MZ1, and DSPE‐PEG_2k_ is rapidly mixed with PBS to formulate LND‐MZ1 at 40 °C. b) Representative TEM image of the LND‐MZ1 via 2% uranyl acetate negative staining. Scale bar = 50 nm. Dynamic light scattering histogram and zeta potential of c) LND‐MZ1 and d) the stored LND‐MZ1 after 45 d at 4 °C. e) HPLC chromatograms and f) quantitative percentage curve of the released MZ1 from 100 µm LND‐MZ1 by incubation with 40 mm DTT for different times within 30 h at 37 °C. The detection wavelength was 254 nm. The data are presented as mean ± s.d. *n* = 3.

To construct an excellent LND‐based nanodelivery efficiently and steadily, a microfluidic device was applied for LND‐MZ1 formulation by controlling the rate ratio of PBS/ethanol as 3/1 at 40 °C (**Figure** **2**a). Initially, PE‐S‐S‐MZ1 was mixed with Hydro Soy PC (HSPC) and 1, 2‐distearoyl‐sn‐glycero‐3‐phosphoethanolamine‐N‐[methoxy(polyethylene glycol)‐2000] (ammonium salt) (DSPE‐PEG_2k_) as LND‐MZ1 formulations. HSPC as high melting temperature (*T*_m_) phospholipids and DSPE‐PEG_2k_ modified with polyethylene glycol could enable LND‐MZ1 with stability and long circulation time in vivo.^[^ ^25^ ^]^ Finally, the molar ratio of PE‐S‐S‐MZ1/HSPC/DSPE‐PEG_2k_ was 1/15/4, which was dissolved in ethanol to rapidly mix with PBS at 40 °C for formulating ideal LND‐MZ1.

To characterize the structure of LND‐MZ1, the morphology of LND‐MZ1 was confirmed by transmission electron microscopy (TEM) via 2% uranyl acetate negative staining. As exhibited in Figure 2b, the LND‐MZ1 presents a disk‐like topology structure with monodispersity and a lipid thickness of 3.1 nm. The LND distribution is consistent and the size distribution of LND‐MZ1 derived from TEM images was measured with a particle size of 97.4 ± 16.9 nm (Figure S9a,b, Supporting Information). The hydrated diameter of the LND‐MZ1 was 126.9 nm with a polydispersity index (PDI) of 0.18 and a zeta potential of ‐5.09 mV (Figure 2c). After 45 d of storage at 4 °C, the hydrated diameter of the LND‐MZ1 was 119.6 nm with a PDI of 0.16 and a zeta potential of ‐4.62 mV (Figure 2d). The differences in size and potential are both within acceptable limits, revealing the excellent stability of LND‐MZ1. Liposomes, containing cholesterol as the main component to stabilize the nanostructures, are common carriers for drug delivery.^[^ ^25^ ^]^ To further verify the performance of LND as a carrier, we synthesized liposomes and compared their morphology and particle size to LND. Validated by TEM and dynamic light scattering (DLS) analysis, LNDs as MZ1 carriers have prominent advantages in dimensional consistency and loading capacity compared with liposomes as carriers of MZ1 with different proportions (Figure S10, Supporting Information).

After demulsification with methanol, the encapsulation efficiency of LND‐MZ1 was tested by HPLC. According to the standard curve of PE‐S‐S‐MZ1 (Figure S8, Supporting Information), the encapsulation efficiency of LND‐MZ1 was calculated to be 48.0%. And then, the release of MZ1 from LND‐MZ1 was also investigated and quantified by HPLC (Figure 2e). After a reaction time of 2 h, mainly intermediates (MZ1‐SH) were produced followed by the release of the final product (MZ1) likewise. Within 30 hours, almost all MZ1 was released from LND‐MZ1 (release efficiency was 98.14%, Figure 2f), further demonstrating the feasibility of releasing MZ1 from LND‐MZ1 in vitro. In addition, we used GSH (10 mm) to verify the release efficiency of LND‐MZ1 at 37 °C in PBS (pH 7.4) as physiologically relevant conditions. As shown in Figure S11 (Supporting Information), MZ1 (*R*_T_ = 25 min) and MZ1 intermediates (MZ1‐SH, *R*_T_ = 27 min) appeared after the GSH treatment of 24 h, demonstrating that MZ1 could be released under the GSH‐abundant tumor microenvironment.

### 2.3 Cellular Internalization Inhibition and Deep Tumor Penetration of LND In Vitro

**Figure 3:**
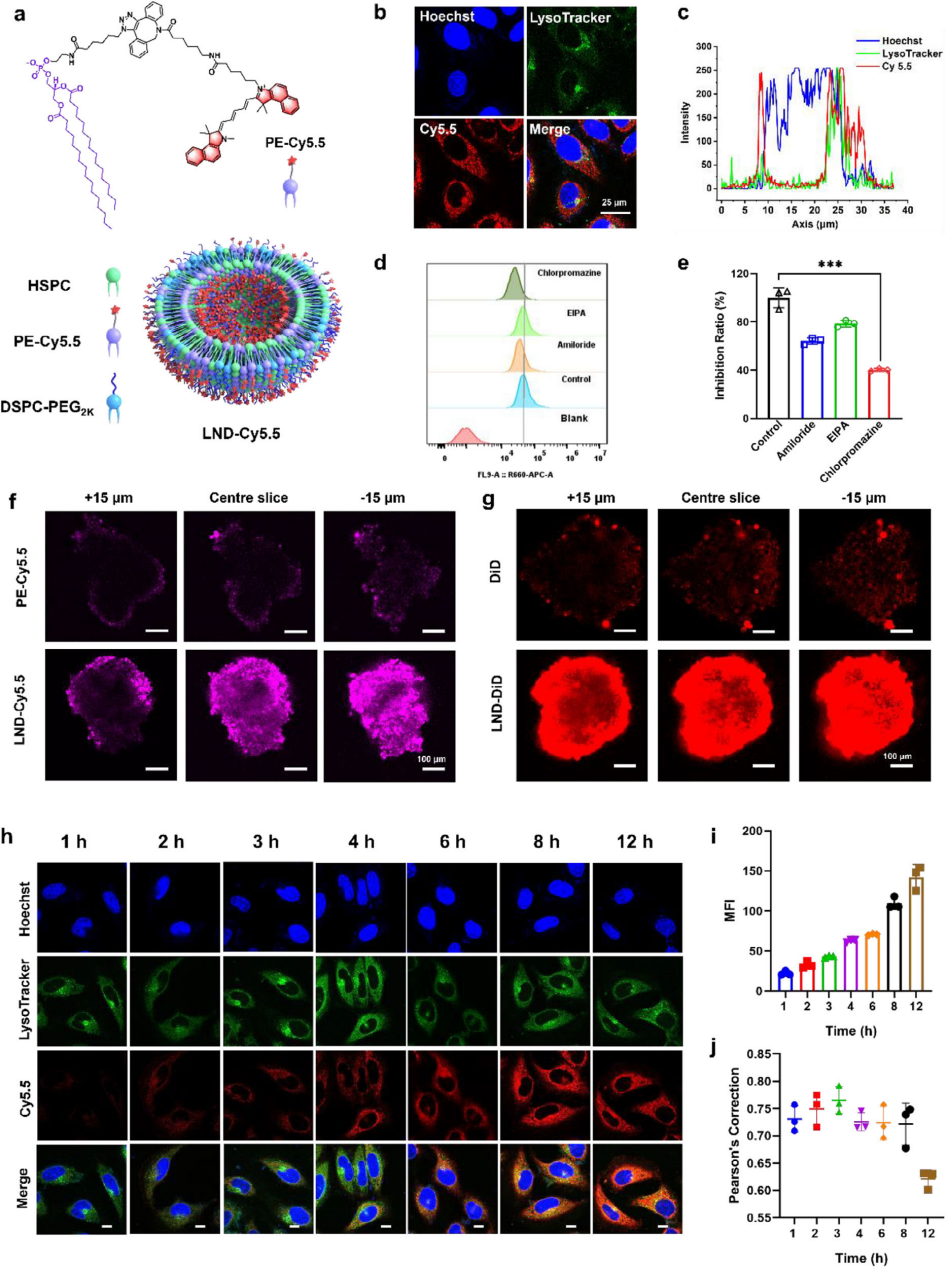
Visualization of cellular internalization inhibition and deep tumor penetration. a) PE‐Cy5.5 synthesized by click chemistry as a substitute of PE‐S‐S‐MZ1 for LND‐Cy5.5 formulation with HSPC and DSPE‐PEG_2k_. b) CLSM images of MCF‐7 cells after the incubation of LND‐Cy5.5 (5 µm) and LysoTracker. Scale bar = 25 µm. c) Intensity profiles of Hoechst, LysoTracker and Cy5.5 signal along the line in (b). d) Flow cytometry and e) quantification of MCF‐7 cells treated by different inhibitors for 30 min and LND‐Cy5.5 (5 µm) for 4 h. The data are presented as mean ± s.d. *n* = 3. One‐way analysis of variance (ANOVA) with Tukey's test correction was used in the statistical analysis test, ***P < 0.005. f) LND‐Cy5.5 displayed increased penetration into MCF‐7 MCSs in vitro compared with PE‐Cy5.5 counterpart. Scale bar = 100 µm. g) LND‐DiD with excellent penetration into MCF‐7 MCSs in vitro compared to the lipid‐soluble DiD alone. Scale bar = 100 µm. h) CLSM images of MCF‐7 cells after the incubation of LND‐S‐S‐Cy5.5 (5 µm) for different times. Scale bar = 10 µm. i) The fluorescence intensity of Cy5.5 and j) colocalization between Cy5.5 and endosome or lysosomes at different times after LND‐S‐S‐Cy5.5 incubation.

A Cy5.5‐modified PE conjugate (PE‐Cy5.5) was synthesized by click chemistry (Scheme S2, Figure S12, Supporting Information), which as a substitute for PE‐S‐S‐MZ1 was applied to formulate LND‐Cy5.5 in the same condition with LND‐MZ1 (**Figure** **3**a). To study the internalization of LND more visually, LND‐Cy5.5 was employed for flow cytometry and confocal laser scanning microscopy (CLSM) test. As shown in Figure S13 (Supporting Information), the LND‐Cy5.5 showed an apparent fluorescence signal of Cy5.5 in MCF‐7 cells compared with the PE‐Cy5.5 group, verifying the superior internalization. In detail, LND‐Cy5.5 was rapidly distributed in the lysosome within 4 hours (Figure 3b,c). To further investigate the underlying mechanisms of LND internalization, we treated the cells with different endocytosis inhibitors. Upon the MCF‐7 cells treated with different inhibitors, such as amiloride (an inhibitor of epithelial sodium channel), EIPA hydrochloride (an inhibitor of macropinocytosis), chlorpromazine (Chlo, an inhibitor of clathrin‐mediated endocytosis), the fluorescence signals of Cy5.5 were weakened in different degrees (Figure 3d). The group treated by Chlo showed the most obvious reduction in fluorescence intensity, which was consistent with the result of CLSM (Figure S14, Supporting Information). By qualifying the flow cytometry results, the fluorescence signal of the group treated by Chlo decreased to 40.3% compared with the control group (Figure 3e), further verifying that LND‐Cy5.5 is taken up by MCF‐7 cells mainly in the clathrin‐mediated endocytosis way.^[^ ^26^ ^]^

Furthermore, the MCF 3D multicellular spheroids (MCSs) were prepared to simulate solid tumors and further evaluate tumor penetration of LND‐Cy5.5. As shown in Figure 3f, the fluorescence signal of Cy5.5 remains obvious in the center of MCF‐7 MCSs, in contrast, the PE‐Cy5.5 only remains in the extension of MCF‐7 MCSs, indicating that the LND as a carrier could improve the solubility of PE‐Cy5.5 and assist PE‐Cy5.5 penetrate the inner of the tumor. Meanwhile, DiD, a commonly used carbocyanine dye for lipophilic tracer, was encapsulated in LND as LND‐DiD to further verify the excellent cell internalization capacity of LND. Compared with lipid‐soluble DiD, LND‐DiD infiltrate into the interior of MCF‐7 MCSs (Figure 3g), which is in consistent with the cellular internalization (Figure S15 Supporting Information), further highlighting the excellent cellular uptake and tumor penetration performance of LND.

To investigate the endocytosis efficiency and endosomal escape of LND‐MZ1, PE‐S‐S‐Cy5.5 was synthesized by conjugating Cy5.5 with phosphatidylethanolamine (PE) via a disulfide bond (Scheme S3, Figure S16, Supporting Information), which was further employed to prepare LND‐S‐S‐Cy5.5. The LND‐S‐S‐Cy5.5 was used for CLSM and colocalization analysis between Cy5.5 and endosome or lysosome in MCF‐7 cells. As shown in CLSM images (Figure 3h,i), the fluorescence intensity of Cy5.5 was increased over the incubation time of LND‐S‐S‐Cy5.5, demonstrating the continuous internalization of LND‐S‐S‐Cy5.5 by cells. By quantifying the colocalization between Cy5.5 and endosome or lysosome, LND‐S‐S‐Cy5.5 localized in endosome or lysosome after endocytosis and the highest level occurred around 3 hours after incubation (Figure 3j). And then, the colocalizations gradually decreased with the prolonged incubation period, which revealed that Cy5.5 escaped from the endosome and lysosome after the incubation for 3 h. As a result, the rapid lysosomal entrapment occurred within endocytosis for only 3 hours, which could not limit the release rate and the efficacy of the drug. The results showed that LND as a carrier was useful for the endocytosis of LND‐MZ1 and the endosomal escape of MZ1 under the redox environment of tumor cells.

### 2.4 BRD4 Degradation and Cell Cytotoxicity of Stimuli‐Activatable LND‐MZ1

**Figure 4:**
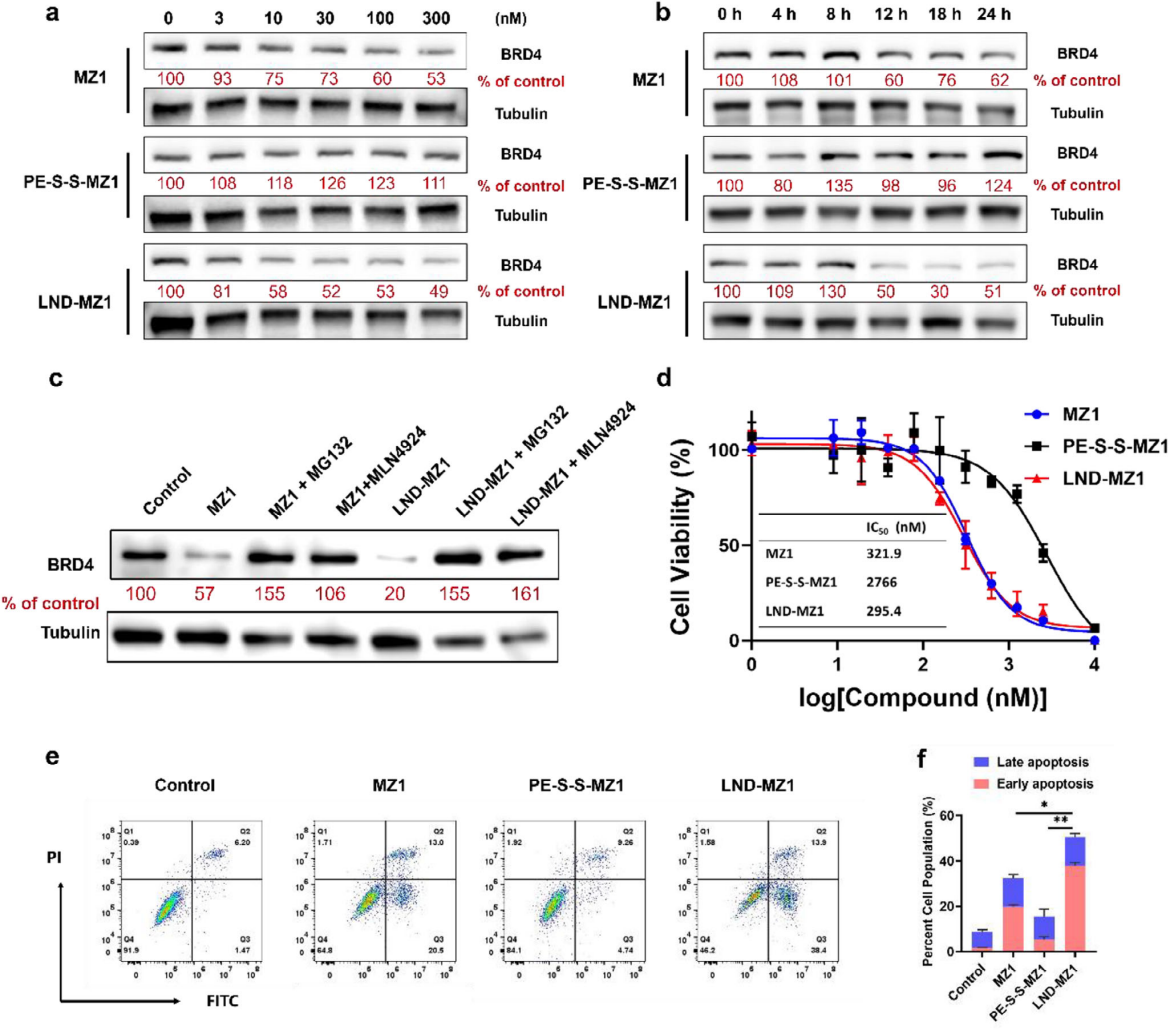
Effects of LND‐MZ1 on reducing the target protein levels and inhibiting cell growth. Western blotting analysis of BRD4 protein degradation a) after 24 h incubation with different drugs and b) after different incubation times with the identified concentration (300 nm). c) Western blotting analysis of BRD4 protein levels of MCF‐7 cells after pretreatment by proteasome inhibitor (10 µm MG132 or 1 µm MLN4924) for 12 h and following treatment by 300 nm MZ1 or LND‐MZ1 for 24 h. d) Cell viability of MCF‐7 after incubation with different drugs at 37 °C for 72 h. The data are presented as mean ± s.d. (*n* = 3). e) Apoptosis detection of MCF‐7 cells treated with different drugs for 24 h with the same concentration (300 nm) by Annexin V‐FITC Kit. f) The quantification of late and early apoptosis. The data are presented as the mean ± s.d. *n* = 3.

To identify the BRD4 degradation performance, the BRD4 levels of MCF‐7 cells after the treatment of different drugs were evaluated by western blotting. In **Figure** **4**a, it is observed that the BRD4 level was dependent on the MZ1 concentration, which is consistent with reports.^[^ ^19b^ ^]^ In contrast, the protein degradability of PE‐S‐S‐MZ1 was limited because of the poor water solubility and membrane permeability. Compared to MZ1, the protein degradation of BRD4 was more evident after the treatment of LND‐MZ1 with the identical concentration of PE‐S‐S‐MZ1, further indicating that LND‐MZ1 facilitated more PROTAC to penetrate cells and enabled the efficient protein degradation of the released MZ1 after activation. The protein degradation abilities of MZ1 and LND‐MZ1 over time also were examined. The BRD4 levels were also dependent on the incubation time for MZ1 and LND‐MZ1 groups (Figure 4b). Notably, a faster reduction in BRD4 levels was observed after LND‐MZ1 incubation compared to that of MZ1, which mainly benefits from that the nanodelivery can be quickly endocytosed by tumor cells and rapidly release PROTAC in high GSH conditions. To further explore the intrinsic‐dependent degradation mechanism of LND‐MZ1, two inhibitors (MG132,^[^ ^27^ ^]^ a proteasome inhibitor, and MLN4924,^[^ ^28^ ^]^ a cullin‐based E3 ligase inhibitor) were applied to pretreat the cells before the drug treatment. In the presence of MG132 or MLN4924, the degradation effects of both MZ1 and LND‐MZ1 were inhibited. The increase in BRD4 levels after treatment with drugs following MG132 or MLN4924 pretreatment could be attributed to an inhibited state of the proteasome and the increased abundance of cullin‐based E3 ligase substrates.^[^ ^27^, ^28^, ^29^ ^]^ These results indicate that the active ingredient of LND‐MZ1 is the released MZ1, which degrades BRD4 relying on E3 ubiquitin ligase in a proteasome‐dependent manner (Figure 4c). In addition, the immunofluorescence staining images of MCF‐7 cells show that the protein degradability of LND‐MZ1 is comparable to that of MZ1, in contrast to the control and PE‐S‐S‐MZ1 groups (Figure S17, Supporting Information).

To assay the cytotoxicity in vitro, the cell viability and half inhibition concentrations (IC_50_) of MCF‐7 treated with different drugs were examined by a cell counting kit‑8 (CCK‐8). In all groups, the cell viability was getting lower and lower with the increasing concentrations. In the graph of Figure 4d, the IC_50_ of LND‐MZ1 (295.4 nm) is slightly lower than the MZ1 group (321.9 nm), and much lower than the PE‐S‐S‐MZ1 group, indicating that LND‐MZ1 comparably inhibits the proliferation of MCF‐7 cells. Meanwhile, the annexin V‐FITC analysis exhibits that LND‐MZ1 evokes cell apoptosis effectively, which is comparable to that of the MZ1 group (Figure 4e,f). Subsequently, the Calcein/PI cell viability test also suggests the LND‐MZ1 induces the most cell death among all the groups (Figure S18, Supporting Information). Collectively, these results highlight that LND has excellent PROTAC delivery ability, enabling more drugs with poor cell permeability to enter tumor cells, and further release active MZ1 to degrade BRD4, resulting in outstanding cell proliferation suppression.

### 2.5 Tumor Penetration and Retention Performance In Vivo

**Figure 5:**
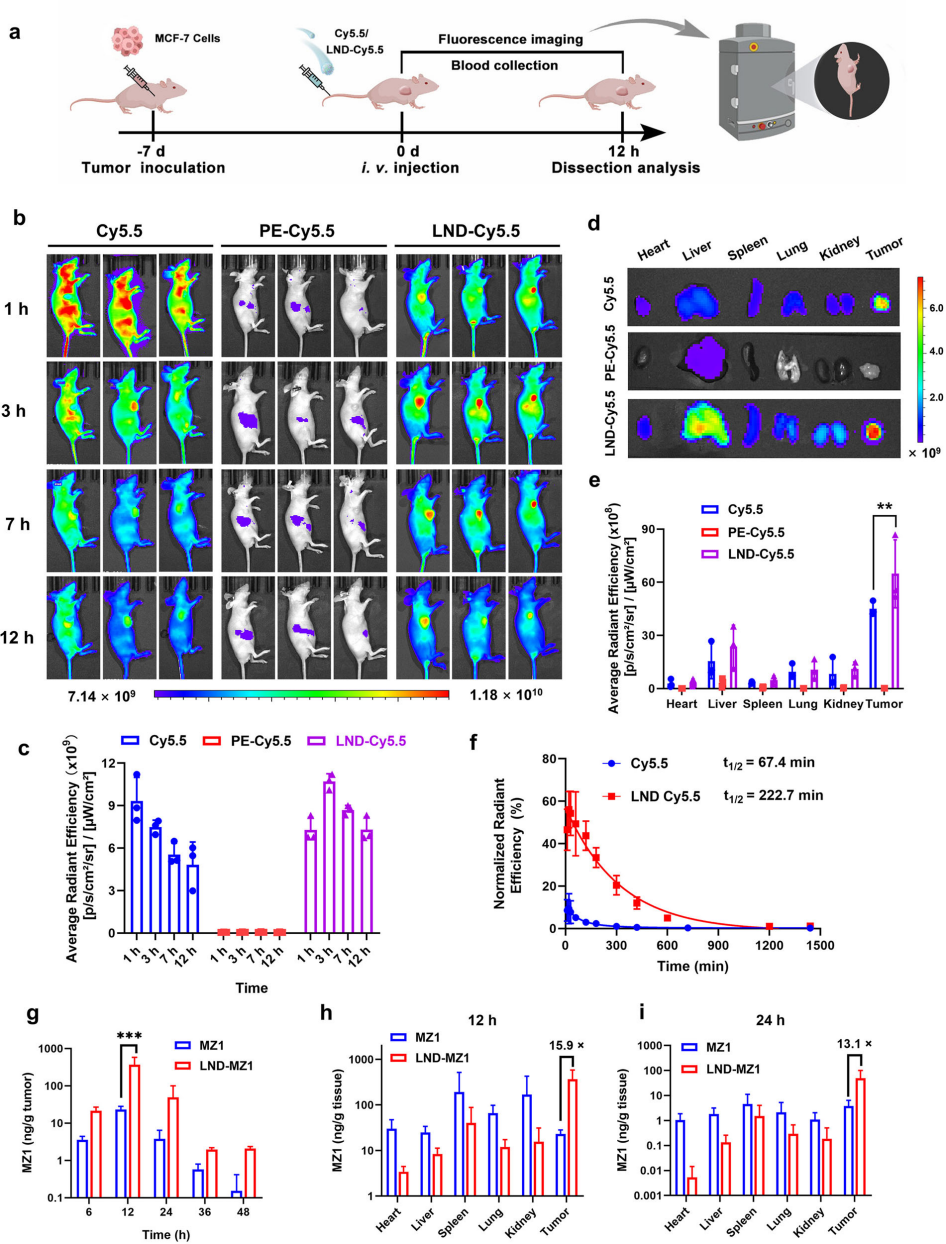
Verification of tumor penetration performance for LND‐Cy5.5 in vivo. a) Schematic of the schedule for the tumor penetration study in tumor‐bearing mice. b) Fluorescence images of Cy5.5 distribution and c) fluorescence quantification of the tumor sections in MCF‐7 tumor‐bearing mice in vivo after the incubating time of 1 h, 3 h, 7 h, 12 h. Mice were i.v. injected with Cy5.5, PE‐Cy5.5, and LND‐Cy5.5 at an Cy5.5 dose of 1 mg kg^−1^ (*n* = 3 biologically independent samples). d) Ex vivo fluorescence images and e) quantification of the major organs and tumors, which were dissected at 12 h post‐injection from tumor‐bearing mice. Statistical significance was calculated via a two‐tailed Student's t‐test, **P < 0.01. f) Normalized radiant efficiency of the collected blood from Cy5.5 and LND‐Cy5.5 administrated mice over time (*n* = 5 biologically independent mice). g) MZ1 concentrations determined by UPLC‐MS in the tumors of mice treated with MZ1 or LND‐MZ1 at different time points (*n* = 3 biologically independent mice). Statistical significance was calculated via a two‐way ANOVA, ***P < 0.005. Biodistribution of MZ1 within the different tissues from MZ1 or LND‐MZ1 administrated mice at h) 12 h and i) 24 h (*n* = 3 biologically independent mice).

The biodistribution performance of the nanodelivery in vivo was examined by fluorescence imaging. MCF‐7 cells were injected subcutaneously into the right flank of BALB/c nude mice. When the tumor volume of mice reached 100 mm^3^, LND‐Cy5.5 and parent Cy5.5 were injected intravenously (i.v.) into mice at an identical dose of 1 mg kg^−1^ (**Figure** **5**a). After the injection for 1 h, the Cy5.5 quickly spread throughout the mice, and then the fluorescence gradually diminished over time, mainly due to the outstanding water solubility and membrane permeability of Cy5.5 (Figure 5b,c). Of note, LND‐Cy5.5 specifically accumulated at the tumor site after the i.v. injection for 1 h and maintained for over 12 h in contrast to PE‐Cy5.5 with poor water solubility. At 12 h post‐injection, the major organs (heart, liver, spleen, lung and kidney) and tumor tissues were collected for *ex vivo* fluorescence imaging. The results displayed excellent tumor distribution of the LND‐Cy5.5, which is more obvious than Cy5.5 and PE‐Cy5.5 (Figure 5d,e).

To analyze the pharmacokinetics (PK) difference between LND‐Cy5.5 and Cy5.5 in mice after i.v. injection, venous blood samples at various time points were collected to measure the fluorescence intensity. A longer blood retention time of LND‐Cy5.5 with a half‐life time of 222.7 min was obtained compared to Cy5.5 with a half‐life time of 67.4 min (Figure 5f), suggesting that LND as a nanodelivery shows a dramatic blood retention capacity to prevent drugs from being cleared too quickly. Overall, the fluorescence results verify that the LND exhibits remarkable tumor‐specific penetration and accumulation, as well as extended pharmacokinetics for site‐specific drug delivery in vivo.

To evaluate the tumor penetration and retention of LND‐MZ1, we quantified the MZ1 content in tumors of mice at various times after i.v. injection of MZ1 or LND‐MZ1 by UPLC‐MS. In Figure 5g, it is shown that MZ1 could be obviously delivered to the tumor section at 6 h and reached the highest content at 12 h as for both the MZ1 and LND‐MZ1 group, indicating the successful MZ1 release from LND‐MZ1. It was worth noting that the MZ1 content of the tumor sections in the LND‐MZ1 group was all higher than in the MZ1 group at different time points, further demonstrating that LND as the nanodelivery could enhance the MZ1 retention capacity on tumors compared to the pure parent MZ1. In addition, the biodistribution of LND‐MZ1 was evaluated more directly based on the MZ1 content of different tissues by UPLC‐MS. At 12 h after the MZ1 injection, more MZ1 accumulated in other organs in addition to the tumor, demonstrating the systemic toxicity of the parent MZ1 (Figure 5h). However, the LND‐MZ1 can release more MZ1 specifically at the tumor site, even at 24 h after LND‐MZ1 injection (Figure 5i). As a result, LND‐MZ1 can not only reduce the systemic toxicity but also increase the retention time of MZ1 at the tumor site.

### 2.6 Antitumor Therapeutic Efficacy and Biosafety of LND‐MZ1 In Vivo

**Figure 6:**
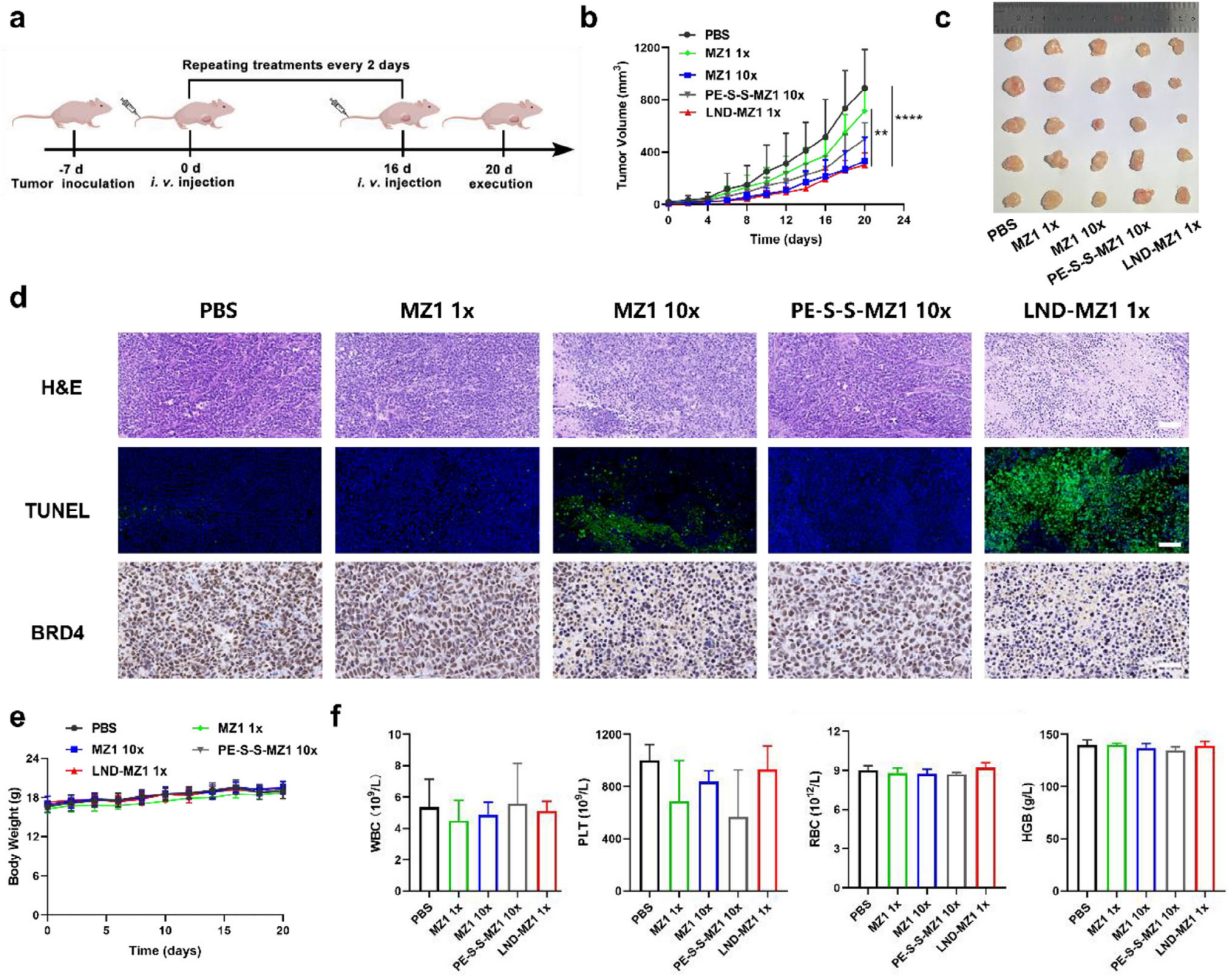
The assessment of antitumor efficacy and biosafety of LND‐MZ1 in the MCF‐7 xenograft mouse model. a) Schematic illustration of the treatment schedule for MCF‐7 tumor‐bearing BALB/c nude mice. From day 0, PBS, MZ1 1× (2 mg kg^−1^), MZ1 10× (20 mg kg^−1^), PE−S‐S‐MZ1 10× (20 mg MZ1 kg^−1^) or LND‐MZ1 1× (2 mg MZ1 kg^−1^) were administered every 2 d via i.v injection. b) Tumor growth curves of the mice upon the treatment of different drugs during 20 d monitoring (*n* = 5 biologically independent mice). One‐way analysis of variance (ANOVA) with Tukey's test correction was used in the statistical analysis test, **P < 0.01, ***P < 0.005. c) Photographs of tumor tissues harvested at day 20. d) H&E, TUNEL staining and IHC examination of dissected tumor tissues upon the treatment of different drugs. H&E, TUNEL staining: scale bar = 100 µm. IHC examination: scale bar = 50 µm. e) Variation curves of the mouse body weight in each group (*n* = 5 biological replicates). (f) Blood routine analyses of mice were performed at day 20 after treatment to assess the toxicity of the LND‐MZ1 (*n* = 4 biologically independent mice).

To explore the antitumor efficacy of LND‐MZ1, different drugs were i.v. injected into tumor‐bearing mice according to the treatment schedule when the tumor volume reached 50 mm^3^ (**Figure** **6**a). After the 20 d treatment cycle, it was worth noting that tumor growth of the LND‐MZ1 group was significantly inhibited at an identical dosage of 2 mg kg^−1^ of MZ1, compared to the MZ1 and PE‐S‐S‐MZ1 groups with the same concentration. Notably, the tumor‐killing effect of the LND‐MZ1 was almost comparable to that of MZ1 at a 10‐fold dose (20 mg kg^−1^), which was attributed to the efficient delivery and precise MZ1 release capabilities of LND‐MZ1 (Figure 6b,c). Moreover, the record of tumor weights further revealed the encouraging antitumor activity of LND‐MZ1 (Figure S19, Supporting Information).

To gain insight into the therapeutic effect in detail, the tissue slices of harvested tumors were used for histopathological analysis (Figure 6d). In hematoxylin‐eosin (H&E) staining, it was observed that the LND 1× and MZ1 10× caused the decrease in the number of tumor cells while no injury areas were found in the other groups. Furthermore, distinct apoptosis signals of LND‐MZ1 1× and MZ1 10× groups in the tumor section also occurred by TUNEL staining, which was consistent with corresponding results in vitro. Additionally, immunohistochemistry (IHC) examinations demonstrated that LND‐MZ1 1× and MZ1 10× played excellent roles in the degradation of the BRD4 protein, verifying that the antitumor efficacy of LND‐MZ1 in vivo was associated with the apoptosis induced by BRD4 degradation. Furthermore, the H&E staining images revealed that different drugs caused almost negligible histopathological damage to the major organs (heart, liver, spleen, lung and kidney) (Figure S20, Supporting Information) and no significant weight loss occurred in all groups (Figure 6e), proving that all drugs had good biosafety, further supporting the therapeutic safety potential of LND‐MZ1. The blood routine analyses of BALB/c nude mice treated for 20 days, including white blood cells (WBC), platelets (PLT), red blood cells (RBC) and hemoglobin (HGB), were executed to assess the toxicity of the LND‐MZ1. In Figure 6f, the index levels of all groups show a little significant difference and remain within acceptable limits. These results suggest that LND‐MZ1 provides precise, robust therapy of tumors and confers no obvious systemic toxicity to mice at the same time.

To further support the broad applicability of LND‐based PROTAC delivery, we further performed an MDA‐MB‐231 xenograft mouse model. When the MDA‐MB‐231 tumor volume reached 50 mm^3^, four groups of BALB/c mice were dosed by i.v. injection with PBS, MZ1 10× (20 mg MZ1 kg^−1^), LND+MZ1 1× (2 mg MZ1 kg^−1^), LND‐MZ1 1× (2 mg MZ1 kg^−1^). After a 16 d of drug treatment (Figure S21a, Supporting Information), changes in tumor volume and body weight of the mice were monitored every 2 d for 32 d. On day 32, tumors were collected and the weights of all tumors were recorded. As shown in Figure S21b‐d (Supporting Information), the tumor suppression effect of LND‐MZ1 was significantly stronger than that of the LND + MZ1 group at the same dose (2 mg kg^−1^), and even stronger than that of the MZ1 group at a 10‐fold dose (20 mg kg^−1^). Additionally, the body weight of the mice in different treatment groups did not show a significant decrease (Figure S21e, Supporting Information), indicating the biosafety of the injected drugs. The results further demonstrated that therapeutic efficacy and broad applicability of LND‐MZ1 on tumors with minimized systemic toxicity.

## 3 Conclusion

In summary, to alter the biophysical properties and minimize the systemic toxicity of PROTAC, we employed the LND‐MZ1 nanodelivery system to deliver MZ1 into tumors specifically, which promoted more MZ1 enter the tumor cell accurately to accelerate protein degradation and extending pharmacokinetics. Our systematic study showed that the LND‐MZ1 entered the tumor cell rapidly and released MZ1 under the intracellular reductive stimulus to perform protein degradation function, suppressing tumor cell proliferation. In vivo, LND‐MZ1 exhibited excellent tumor penetration and retention at the tumor site, as well as an extended blood circulation. Trials of LND‐MZ1 in tumor‐bearing mice further confirmed that LND‐MZ1 was effective in inhibiting tumor growth at a lower dose than the parent MZ1 alone. Meanwhile, LND‐MZ1 showed satisfactory biosafety and minimized the off‐target toxicity of PROTAC in vivo. The LND is a versatile tool for PROTAC delivery to elicit pathogenic protein degradation and provides new opportunities to explore LND‐based delivery systems for other potential drugs with limited physicochemical and pharmacokinetic properties.

## 4 Experimental Section

### Preparation of LND‐MZ1

LND‐MZ1 was formulated in a microfluidic device (Micro&Nano Biologics Co., Ltd., China) by mixing PBS with ethanol phases containing the ionizable lipid. The PBS was heated to 40 °C in advance and loaded into a syringe. HSPC (29.4 mg, 0.0375 mol), DSPE‐PEG_2k_ (28.0 mg, 0.01 mol), PE‐S‐S‐MZ1 (5.8 mg, 0.0025 mol) at the ratio of 15/4/1 were diluted in ethanol (3 mL) and heated to 40 °C for dissolution. The lipid solution that made up the nanoparticles also was loaded into a second syringe. The PBS and ethanol phases were mixed in an aqueous to ethanol ratio of 3/1 by volume at a total rate of 12 mL min^−1^ at 40 °C using syringe pumps, then incubated for 10 min at room temperature. LND‐MZ1 solution was dialyzed against 1× PBS in a 20 kDa MWCO cassette (Millipore, USA) for 3 h, filtered through a 0.22 µm filter (JINTEN, China) and stored at 4 °C. The size and zeta potential of the purified LND‐MZ1 were measured by the Zetasizer Nano (Nano‐ZS, Malvern Panalytical). The morphology of the purified LND‐MZ1 was characterized by the TEM (H‐7650B, Hitachi). The PE‐S‐S‐MZ1 content of the purified LND‐MZ1 was measured by HPLC and was calculated by the standard curve of PE‐S‐S‐MZ1, when the LND‐MZ1 was completely demulsified in methanol by sonication for 10 min. The obtained PE‐S‐S‐MZ1 content of the purified LND‐MZ1 was used as the concentration of LND‐MZ1.

When noted, fluorescent labeled LNDs were prepared in a similar way to LND‐MZ1 by replacing PE‐S‐S‐MZ1 with PE‐Cy5.5 or PE‐S‐S‐Cy5.5, which was named as LND‐Cy5.5 or LND‐S‐S‐Cy5.5. The concentration of LND‐Cy5.5 was calculated according to the parent PE‐Cy5.5 of formulation. LND‐DiD was prepared by adding DiD (100 mm, 0.25 µL) to the lipid content. The other lipid formulation and operational process were the same as that of LND‐MZ1. The concentration of LND‐DiD was calculated according to the parent DiD of formulation. LND + MZ1 was prepared in a similar way to LND‐MZ1 by replacing PE‐S‐S‐MZ1 with PE‐N_3_ and further adding MZ1 into ethanol.

### Drug Release Kinetics of PE‐S‐S‐MZ1 and LND‐MZ1

The HPLC analysis was obtained by the column (Ultimate XB‐C8, 4.5 × 150 mm, 5 µm, 300 Å, Welch). The drug release kinetics studies of PE‐S‐S‐MZ1 and LND‐MZ1 were carried out using DTT (40 mm, pH = 7.2–7.4) or GSH (10 mm, pH = 7.2–7.4) solution as the release medium in a 5 mL bottle. The reaction was maintained at 37 °C and kept stirring at 100 rpm. At various time points, 40 µL of solution was got out to mix with 120 µL methanol by sonication for 10 min. The completely dissolved mixture was analyzed by HPLC to quantify the contentment of the released MZ1 [eluent A: H_2_O (0.1% TFA); eluent B: ACN (0.1% TFA), rang from 10% B (at min 5) to 100% B (at min 30) and keep for 10 min]. The detection wavelength was 254 nm.

### Cell Viability Assays

MCF‐7 cells were seeded at 5 × 10^3^ cells per well in 96‐well plates for 24 h before treatment. Then, the cells were treated with MZ1, PE‐S‐S‐MZ1 or LND‐MZ1 in quadruplicate or bipartite for each concentration point. After 72 h treatment, the cell viability was measured with a cell counting kit‑8 (CCK‐8, Solarbio) according to the manufacturer's instructions. The absorption at 450 nm was determined with a plate reader (EnVision, Perkin). Data were analyzed with GraphPad Prism software to obtain the IC_50_ values of each test group.

The MCF‐7 cells were seeded at 1 × 10^5^ cells per well in 12‐well plate for 24 h before treatment. After 24 h treatment with different drugs (MZ1, PE‐S‐S‐MZ1 or LND‐MZ1) at the identical concentration of 300 nm MZ1 or PE‐S‐S‐MZ1, cells were washed with ice‐cold PBS and digested with 0.25% trypsin solution without EDTA. Then, the apoptosis of cells was detected by flow cytometry (FACSymphony S8, BD) according to the manufacturer's instruction of ANNEXIN V‐FITC/PI Apoptosis Detection Kit (Solarbio).

The MCF‐7 cells were seeded at 1 × 10^5^ cells per well in confocal dishes (35 mm, Solarbio) for 24 h before treatment. The cells were pretreated with different drugs as described above, and then were stained by Calcein/PI Cell Viability/Cytotoxicity Assay Kit viability (Beyotime) according to the manufacturer's instruction. And then, images were taken using a confocal laser scanning microscope (CLSM, SP8 STED super‐resolution confocal microscope, Leica).

### Western Blotting

MCF‐7 cells were seeded at 5 × 10^5^ cells per well in 6‐well plates for 24 h before treatment. After treatment with different drugs, cells were washed with ice‐cold PBS and scraped off with a cell brush on ice. Then, the cells were collected by centrifugation and lysed in ice‐cold RIPA lysis buffer (containing 1% protease inhibitors Cocktail II). Lysate was vortexed on ice and the supernatant as lysis product was collected by centrifugation at 11 000  *rpm* for 15 min at 4 °C. Protein concentrations were measured by BCA Protein Assay Kit (EpiZyme, Shanghai, China). The collected proteins were added to 5 × loading buffer (Yeasen) and denatured by heating to 100 °C for 15 min. And then, proteins were resolved in 4–12% Bis‐Tris SurePAGE gel (Genscript) and transferred to 0.45 µm PVDF membrane (Millipore) for blotting. The membrane was blocked by QuickBlock blocking buffer (Beyotime) for 20 min at room temperature. The PVDF membrane was incubated in anti‐BRD4 (1:500; Abcam, ab128874), or anti‐tubulin (1:1000; Immunoway, YT4780) overnight at 4 °C. The obtained membrane was then incubated in goat anti‐rabbit HRP secondaryantibodies (1:5000; Immunoway, RS0002) for 1 h at room temperature with shake. All the processes of PVDF incubation were performed on eZwest Pro Automated Western Blotting System (Genscript). After being washed with TBST, the bands were imaged on a GelView 6000Plus System (BLT Photon Technology) with BeyoECL Moon developer (Beyotime). The relative expressions of BRD4 were quantified by Image J with normalization to tubulin and the untreated samples per group.

### Immunofluorescence

Glass coverslips (NEST, 15 mm diameter) were preplaced in 12‐well plates. 1 × 10^5^ MCF‐7 cells were seeded in each well and cultured for 24 h for cell attachment. Then the cells were incubated with DMEM, 300 nm MZ1, 300 nm PE‐S‐S‐MZ1 or 300 nm LND‐MZ1 for 24 h. The coverslips were washed three times with ice‐cold PBS, followed by fixation in 4% paraformaldehyde for 15 min, permeation with 0.5% Triton X‐100 for 20 min, blocking with QuickBlock Blocking Buffer (Beyotime, P0102) for 1 h. And then, the coverslips were immunostained with anti‐BRD4 (1:100, Abcam, ab128874) for 2 h, Alexa Fluor 488 labeled goat anti‐rabbit IgG(H+L) (1:500, Beyotime, A0423) for 2 h successively. Ultimately, after three washes by Immunol Staining Wash Buffer (Beyotime, P0106), the coverslips were covered on slides using Dapi‐Fluoromount‐G (SouthernBiotech). Fluorescence images were obtained by CLSM examination.

### Visualization of In Vitro Cell Delivery and Deep Tumor Penetration

To investigate cellular uptake of the LND, MCF‐7 cells cultured in confocal dishes were incubated with PE‐Cy5.5 and LND‐Cy5.5 at the identical PE‐Cy5.5 concentration of 5 µm for 8 h. The intracellular distribution of the LND nanoparticles was investigated by CLSM (SP8 STED super‐resolution confocal microscope, Leica).

Furthermore, 3D multicellular spheroids (MCSs) of the MCF‐7 were employed to illustrate the tumor penetration profiles of the LND in vitro visually. Briefly, the 3D tumor spheroids were prepared by culturing MCF‐7 cells in 96‐well tissue culture plate (5000 cells per well) which was pretreated with 1% agarose gel. After the 7 d of growth, the 3D tumor spheroids were then incubated with the PE‐Cy5.5 and LND‐Cy5.5 for 8 h. Nanoparticle distribution inside the MCF‐7 MCSs was then investigated by CLSM examination.

### Cellular Internalization Inhibition

MCF‐7 cells were seeded in a 12‐well plate at a density of 1 × 10^5^ per well overnight. Cells were pretreated with 50 µm EIPA, 500 µm amiloride, and 50 µm chlorpromazine for 30 min. Then, LND‐Cy5.5 was added to the mixed solution to keep the concentration of PE‐Cy5.5 at 5 µm and incubate for 4 h continually. The cells were washed with PBS, digested with 0.25% trypsin solution and dispersed in PBS. The fluorescence intensities of the treated cells were measured by flow cytometry (CytoFLEX LX, Beckman Coulter).

The MCF‐7 cells were seeded at 1 × 10^5^ cells per well in confocal dishes (35 mm, Solarbio) for 24 h before treatment. The cells were pretreated by different inhibition drugs as described above and stained by LND‐Cy5.5 for 4 h. Nuclei and lysosomes were stained with 1 × Hoechst 33342 (Beyotime) and LysoTracker (50 nm, Invitrogen) before images were taken by CLSM.

### Endosomal Escape of LND

After the incubation of LND‐S‐S‐Cy5.5 (5 µm) for different times, the MCF‐7 cells were stained by Hoechst 33342 and LysoTracker for 15 min to locate the nuclei and lysosomes, respectively. The fluorescence intensity and colocalization analysis was performed by using software ImageJ.

### Biodistribution Studies

All the animal experiments were approved by the Animal Ethics Committee of Tsinghua University (Approve no. 24‐LJH3). To visualize LND delivery in vivo, subcutaneous MCF‐7 tumor‐bearing mice (*n* = 3, ≈100 mm^3^) were intravenously injected with Cy5.5 (1 mg kg^−1^), PE‐Cy5.5 (2 mg kg^−1^), LND‐Cy5.5 (2 mg PE‐Cy5.5 per kg). Mice were imaged at 1, 3, 7, and 12 h post i.v. injection (excitation at 660 nm; emission at 710 nm). At 12 h, heart, liver, spleen, lung, kidneys and tumors were imaged using the LuminaIII system (PerkinElmer, Waltham, MA, USA) equipped with Living Image software.

### Pharmacokinetics Analysis

To evaluate the effect of LND delivery more directly, MCF‐7 tumor‐bearing mice (*n* = 3, ≈200 mm^3^) were intravenously injected with MZ1 (2 mg kg^−1^) or LND‐MZ1 (2 mg MZ1 kg^−1^). The MZ1 content in tissues (e.g., heart, liver, spleen, lung, kidney and tumor) at 12 and 24 h i.v. injection and in tumors of different times (e.g., 6 h, 12 h, 24 h, 36 h, and 48 h) were harvested and ground, which were further extracted with methanol for MZ1 UPLC‐MS measurement. The UPLC‐MS measurement was completed by Center of Pharmaceutical Technology of Tsinghua University. The UPLC‐MS was equipped with AB SCIEX ExionLC AD HPLC system and AB SCIEX QTRAP 5500 triple quadrupole‐linear ion trap tandem mass spectrometer.

### Blood Circulation Studies

To analyze the blood circulation difference between LND‐Cy5.5 and Cy5.5 in mice after administration, venous blood samples at various time points were collected into centrifuge tube to measure the fluorescence intensity by the LuminaIII system. Blood normalized radiant efficient (%) was calculated as:

(1) $$\frac{\left(F_{t}-F_{\mathrm{control}}\right)}{\left(F_{0}-F_{\mathrm{control}}\right)}\times 100$$

where *F*_t_ is the fluorescence intensity of collected blood per gram over time, *F*_control_ is the fluorescence intensity of per gram of control blood, *F*_0_ is the fluorescence intensity of the collected blood obtained immediately after injection. The half‐life period was calculated with Prism 8.0 (GraphPad Software) by one phase decay.

### Therapeutic Efficacy Investigation of LND‐MZ1 In Vivo

When the MCF‐7 tumor volume reached 50 mm^3^, five groups of BALB/c mice were dosed by i.v. injection with PBS (10 mm, pH 7.2‐7.4), MZ1 10× (20 mg MZ1 kg^−1^), MZ1 1× (2 mg MZ1 kg^−1^), PE‐S‐S‐MZ1 10× (20 mg MZ1 kg^−1^), LND‐MZ1 1× (2 mg MZ1 kg^−1^). MZ1 and PE‐S‐S‐MZ1 were formulated in 1.25% DMSO, 20% PEG300, 5% Tween 80, and 73.75% H_2_O with 10 min sonication. Changes in body weight and tumor volume of the mice were followed every 2 days for 20 days. Tumor volumes were calculated with the formula: the volume = length ×width^2^/2. On day 20 after the drug injection, blood was withdrawn by orbital extraction and major organs (e.g., heart, liver, spleen, lung, kidney and tumor) were collected. The weights of all tumors were recorded. Blood was collected in EDTA K2 blood routine tubes (Wuhan Saville) and was sent to Wuhan Saville Biotechnology Co., Ltd for blood routine tests.

100 µL MDA‐MB‐231 cells (1 × 10^7^ cells, PBS: Matrigel = 1:1, v/v) were injected subcutaneously into the right flank of BALB/c nude mice. When the MDA‐MB‐231 tumor volume reached 50 mm^3^, groups of four BALB/c mice were dosed every 2 days by i.v. injection with PBS (10 mm, pH 7.2–7.4), MZ1 10× (20 mg MZ1 kg^−1^), LND + MZ1 1× (2 mg MZ1 kg^−1^), or LND‐MZ1 1× (2 mg MZ1 kg^−1^) for a 16 d of drug treatment. Changes in body weight and tumor volume of the mice were followed every 2 d for 32 d. On day 32 after the drug injection, tumors were collected and the weights of all tumors were recorded.

### Histological Studies

After 20 d of different treatments, MCF‐7 tumor‐bearing mice in each group were euthanized, then the tumors and major organs (heart, liver, spleen, lung, and kidney) were collected and fixed with 4% paraformaldehyde overnight, and then embedded by paraffin. Immunohistochemistry (IHC) of tumors was obtained from Wuhan Saville Biotechnology Co., Ltd. The TUNEL staining was obtained by the One Step TUNEL Apoptosis Assay Kit (Green, Beyotime) according to the manufacturer's instructions. The hematoxylin‐eosin (H&E) of tissues for histological analysis was obtained from Laboratory Animal Resources Center of Tsinghua University. The stained tissue sections by TUNEL, immunohistochemical and H&E staining were examined on the Pannoramic SCAN II slide scanner (3DHISTECH).

### Statistical Analysis

The level of significance in all statistical analyses was set at *P < 0.05, **P < 0.01, ***P < 0.005, ****P < 0.001. Data are presented as mean ± s.d. and were analyzed using one‐way analysis of variance (ANOVA) with Tukey's test correction in statistical analysis test for groups followed by Tukey's multiple comparisons test using Prism 8.0 (GraphPad Software). Statistical significances of the fluorescence intensity of major organs and tumors were calculated by Two‐way ANOVA.

## Conflict of Interest

The authors declare no conflict of interest.

## Supporting information



Supporting Information

## Data Availability

The data that support the findings of this study are available from the corresponding author upon reasonable request.
