# Supplementary material for: Remodeling the Physicochemical and Pharmacokinetic Properties of PROTAC via Lipid Nanodisks for Cancer Therapy
Source: Adv Sci (Weinh). 2025 Jun 29;12(36):e01384. doi: 10.1002/advs.202501384 (PMC12462925; doi:10.1002/advs.202501384)
Supplement: Supplementary file 1 — Supporting Information [file ADVS-12-e01384-s001.docx]

**Supporting Information**

# Remodeling the Physicochemical and Pharmacokinetic Properties of PROTAC *via* Lipid Nanodisks for Cancer Therapy

Meichen Pan^1,2, †^, Chunrong Yang^2, †^, Zhongliang Fu^1, 2^, Yuchen Yang^2^, Ying Zhuo^3^, and Jinghong Li^2, 4, 5, 6,^ *

1. School of Biomedical Sciences, Hunan University, Changsha, Hunan 410082, China.

2. Department of Chemistry, Center for BioAnalytical Chemistry, Key Laboratory of Bioorganic Phosphorus Chemistry & Chemical Biology, Tsinghua University, Beijing 100084, China.

3. Key Laboratory of Luminescence Analysis and Molecular Sensing (Southwest University), Ministry of Education, College of Chemistry and Chemical Engineering, Southwest University, Chongqing 400715, P. R. China.

4. Beijing Life Science Academy, Beijing 102209, China.

5. New Cornerstone Science Laboratory, Shenzhen 518054, China.

6. Center for BioAnalytical Chemistry, Hefei National Laboratory of Physical Science at Microscale, University of Science and Technology of China, Hefei 230026, China.

E-mail: jhli@mail.tsinghua.edu.cn
Jinghong Li: orcid.org/0000-0002-0750-7352

***Correspondence:** [jhli@mail.tsinghua.edu.cn](mailto:jhli@mail.tsinghua.edu.cn)

^†^M.P. and C.Y. contributed equally to this paper

# **Chemical reagents**

4,4’-Dithiodibutyric acid (95%) was purchased from TCI (Tokyo, Japan). 4-Dimethylaminopyridine (DMAP, 98%), DL-dithiothreitol (DTT, 99%), 1-(3-dimethylaminopropyl)-3-ethylcarbodiimide hydrochloride (EDC, 95%), anhydrous dimethyl formamide (DMF, 98%), and N, N-diisopropylamine (DIPEA, 99.5%) were purchased from Energy-Chemical. (Anhui, China). Hydro Soy PC (HSPC, 98%) was purchased from Aladdin (Shanghai, China). 1, 2-Distearoyl-sn-glycero-3-phosphoethanolamine-N-[methoxy(polyethylene glycol)-2000] (ammonium salt) (DSPE-PEG_2K_) was purchased from Macklin (Shanghai, China). Dibenzocyclooctyne-Cy5.5 (DBCO-Cy5.5, 97.68%), amiloride (Ami, 99.8%), ethylisopropylamiloride (EIPA, 99.73%), chlorpromazine and matrigel (basement membrane matrix HC) were purchased from MedChemExpress (New Jersey, USA). DBCO-S-S-Cy5.5 were purchased from Qiyue Biology (Xian, China). 16:00 azidocaproyl PE (PE-N_3_) was purchased from Kaixin Biotech (Xian, China). 3-Amino-1-(11,12-didehydrodibenz[b,f]azocin-5(6H)-yl)-1-propanone (DBCO-NH_2_, 98%), O-(7-Azabenzotriazol-1-yl)-N,N,N,N-tetramethyl uronium hexafluorophosphate (HATU, 98.8%), (2S,4R)-N-((S)-2-(tert-Butyl)-17-((S)-4-(4-chlorophenyl)-2,3,9-trimethyl-6H-thieno[3,2-f][1,2,4]triazolo[4,3-a][1,4]diazepin-6-yl)-4,16-dioxo-6,9,12-trioxa-3,15-diazaheptadecan-1-oyl)-4-hydroxy-1-(4-(4-methylthiazol-5-yl)benzyl)pyrrolidine-2-carboxamide (MZ1, 98%) were purchased from Bidepharm (Shanghai, China). Dimethyl sulfoxide (DMSO, 99.5%), PBS (10 mM, pH7.2-7.4) and chlorpromazine hydrochloride (Chlo, 98%) were purchased from Solarbio (Beijing, China). PEG300 and protease Inhibitor Cocktail II (100×, DMSO) were purchased from TargetMOI (Shanghai, China). All chemicals were purchased from commercial suppliers and used without further purification. The eluting solvents for high performance liquid chromatography (HPLC) purification were HPLC grade.

# **Chemical synthesis**

The ^1^H NMR spectra were measured on JEOL ECS 400 MHz spectrometer using TMS (0 ppm) as the internal standard and analyzed by MestReNova (v. 12.0.1). The high-resolution mass spectra (HRMS) were detected *via* a quadrupole time-of-flight mass spectrometer with an electrospray ionization (ESI) source (Bruker Daltonics). The mass spectrum of PE-S-S-MZ1 was obtained by matrix-assisted laser desorption/ionization (MALDI) TOF mass spectrometers. The HPLC for purification was obtained by Essentia LC-16P (Shimadzu) with a C8 reversed-phase separation column.


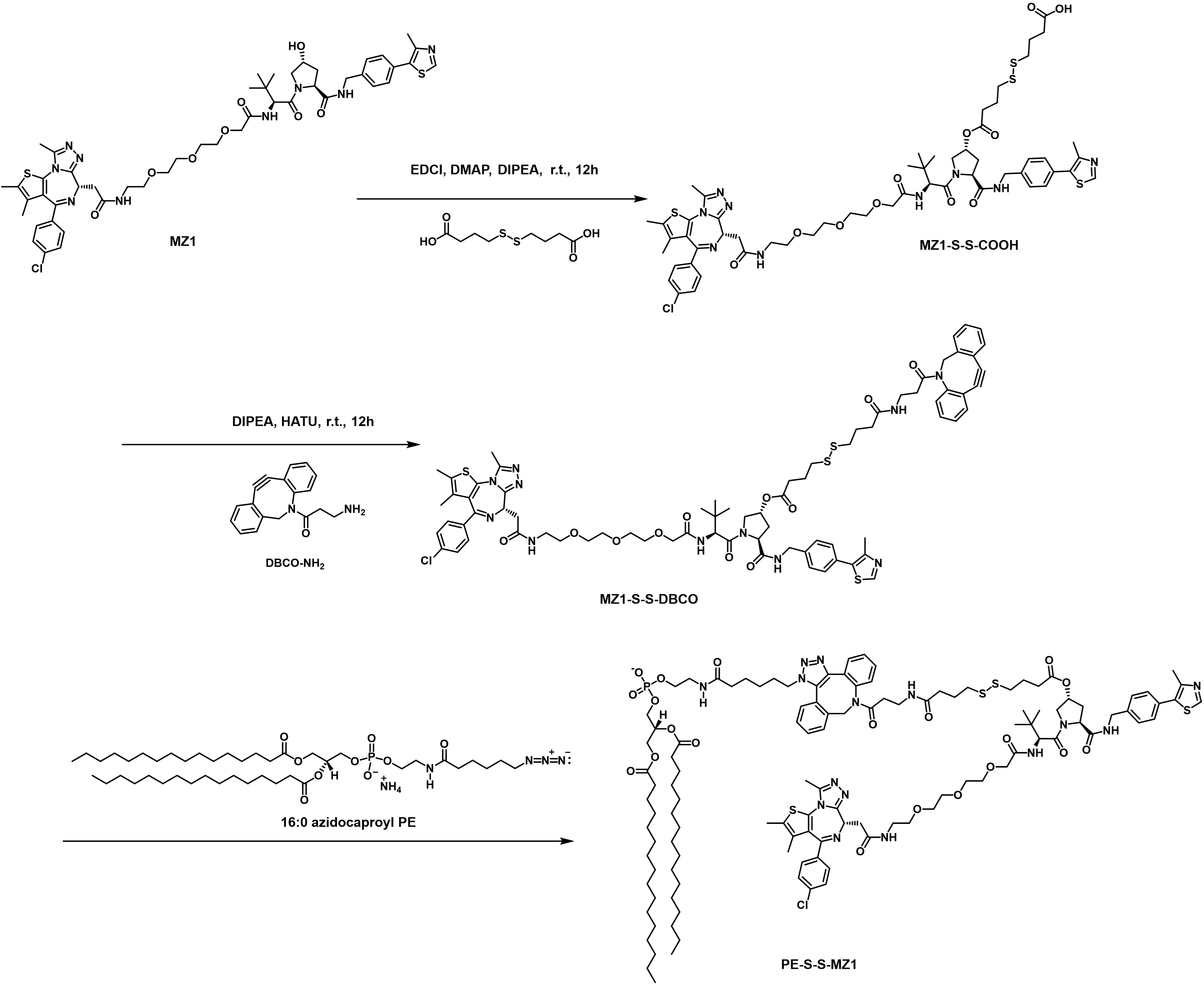


Scheme S1. Reagents and conditions of the synthesis of PE-S-S-MZ1.

## The synthesis of MZ1-S-S-COOH**.**

4,4’-Dithiodibutyric acid (95.34 mg, 0.4 mmol) and DMAP (48.86 mg, 0.4 mmol) were added to the anhydrous DMF (4 mL) solution containing MZ1 (200.6 mg, 0.2 mmol). The mixed solution was stirred at 0 ^o^C, to which EDC (76.68 mg, 0.4 mmol) were added for reaction. The reaction was stirred at room temperature for 12 h and monitored by TLC. The reaction mixture was quenched by the addition of H_2_O (4 mL) and then was extracted with EtOAc (10 mL × 3). The combined organic layers were washed with saturated NaCl solution (10 mL × 3), and dried over Na_2_SO_4_, filtered, and concentrated under reduced pressure to give a residue. The residue was purified by silica gel column chromatography (CH_2_Cl_2_/CH_3_OH = 100/1 to 20/1). MZ1-S-S-COOH was obtained as a yellow solid (124.8 mg, yield 51%). ^1^H NMR (400 MHz, DMSO-*d*_6_) δ 8.98 (s, 1H), 8.65 (t, *J* = 6 Hz, 1H), 8.28 (t, *J* = 5.6 Hz, 1H), 7.48 (d, *J* = 8.7 Hz, 2H), 7.43-7.41 (m, 3H), 7.39 (m, 3H), 5.33-5.29 (m, H), 4.27 (dd, *J* = 15.8, 5.8 Hz, 1H), 3.97-3.94 (m, 3H), 3.83 (dd, *J* = 11.8, 4.1 Hz, 1H), 3.62-3.54 (m, 8H), 3.44 (t, *J* = 5.9 Hz, 2H), 3.28-3.22 (m, 4H), 3.17 (s, 4H), 2.70 (q, *J* = 7.2 Hz, 4H), 2.59 (s, 3H), 2.45 – 2.37 (m, 7H), 2.3-2.24 (m, 3H), 2.16-2.09 (m, H), 2.03-1.95 (m, H), 1.91-1.8 (m, 4H), 1.62 (s, 3H), 0.95 (s, 9H). HRMS (ESI) for C_57_H_72_ClN_9_O_11_S_4_ [M+Na]^+^: calcd.:1244.3815; found: 1244.3810.

## **T**he synthesis of MZ1-S-S-DBCO.

A DMF solution (1 mL) containing MZ1-S-S-COOH (122 mg, 0.1 mmol), DBCO-NH_2_ (27.6 mg, 0.1 mmol), HATU (76 mg, 0.2 mmol), and DIPEA (52.5 μL) was stirred for 12 h at room temperature. After the reaction was completed, the solvent was added H_2_O (2 mL) and then was extracted with EtOAc (4 mL × 3). The combined organic layer was washed with saturated NaCl solution (10 mL × 3), and dried over Na_2_SO_4_, filtered, and concentrated under reduced pressure to afford the crude product. The crude product was purified by silica gel column chromatography (CH_2_Cl_2_/CH_3_OH = 100/1 to 20/1) and the collected solution was dried under reduced pressure to obtain yellow solid product (81.5mg, yield 55%). ^1^H NMR (400 MHz, DMSO-*d*_6_) δ 8.97 (s, 1H), 8.61 (t, *J* = 6 Hz, 1H), 8.27 (t, *J* = 5.7 Hz, 1H), 7.66-7.61 (m, 2H), 7.59-7.56 (m, 1H), 7.50-7.46 (m, 4H), 7.43-7.43 (m, 4H), 7.40-7.38 (m, 6H), 7.37-7.32 (m, 1H), 7.30-7.28 (m, 1H), 5.28 (s, 1H), 5.03 (d, *J* = 12.0 Hz, 1H), 4.52-4.41 (m, 4H), 4.3-4.24 (m, 1H), 3.97-3.95 (m, 3H), 3.64-3.53 (m, 11H), 3.45-3.42 (m, 2H), 3.28-3.20 (m, 3H), 2.7-2.65 (m, 3H), 2.61-2.55 (m, 5H), 2.45-2.42 (m, 4H), 2.40-2.37 (m, 6H), 2.02 (t, *J* = 6 Hz, 2H), 1.89-1.79 (m, 4H), 1.76-1.69 (m, 2H), 1.61 (s, 3H), 0.95 (s, 9H). HRMS (ESI) for C_75_H_86_ClN_11_O_11_S_4_ [M+Na]^+^: calcd.: 1502.4972; found: 1502.4976.

## **The synthesis of PE-S-S-MZ1.**

MZ1-DBCO (14.8 mg, 0.01 mmol) and 16:0 azidocaproyl PE (PE-N_3_,8.4 mg, 0.01 mmol) were added to the solution (CH_2_Cl_2_/CH_3_OH = 1/1, 2 mL) and stirred at room temperature for 12 h (Scheme S1). The reaction solution was concentrated under reduced pressure and was purified by HPLC [eluent A: H2O (0.1% TFA); eluent B: ACN (0.1% TFA), rang from 10% B (at min 5) to 100% B (at min 30) and keep for 10 min]. Yellow solid (22 mg) with 95% yield was concentrated under reduced pressure to attain. MS (MALDI-TOF) for C_118_H_168_ClN_15_O_2_0PS_4_ [M+H]^+^: calcd.: 2311.1011; found: 2312.8412.

PE-Cy5.5 was prepared and purified in a similar way to PE-S-S-MZ1, by replacing the MZ1-DBCO with DBCO-Cy5.5 (Scheme S2). HRMS (ESI) for C_104_H_145_ClN_8_O_11_P [M+Na]^+^: calcd.: 1737.0696; found: 1737.0692.

PE-S-S-Cy5.5 was prepared and purified in a similar way to PE-S-S-MZ1, by replacing the MZ1-DBCO with DBCO-S-S-Cy5.5 (Scheme S3). HRMS (ESI) for C_106_H_148_N_9_O_12_PS_2_ [M+Na]^+^: calcd.: 1858.0353; found: 1858.0351.

Scheme S2. Reagents and condition of the synthesis of PE-Cy5.5.


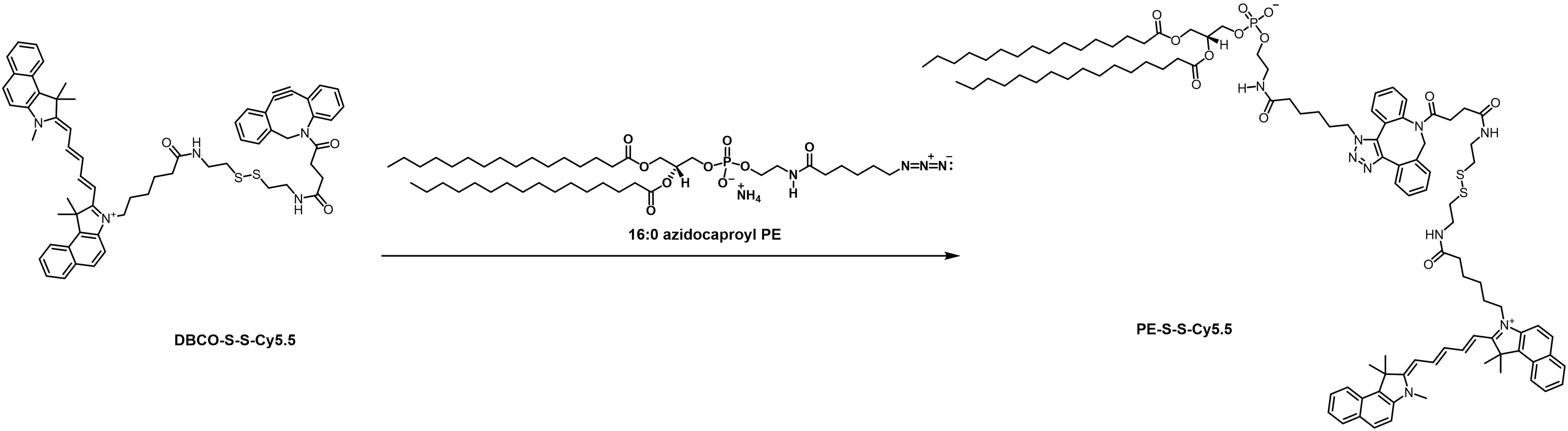


Scheme S3. Reagents and condition of the synthesis of PE-S-S-Cy5.5.

# **Cell and mice**

Trypsin-EDTA solution and penicillin-streptomycin solution were purchased from Yeasen Biotechnology Co., Ltd (Shanghai, China). Dulbecco's Modified Eagle's Medium (DMEM) and fetal bovine serum (FBS) were purchased from Gibco (MD, USA). MCF-7 cells were purchased from the National Infrastructure of Cell Line Resource (NICLR, China). MDA-MB-231 cells were purchased from the Pricella (Wuhan, China). Cells were cultured in DMEM medium containing 10% FBS and 1% penicillinstreptomycin. Cells were cultured in an incubator (Thermo Fisher Scientific) at 37 °C under an atmosphere of 5% CO_2_.

BALB/c nude mice (female; 6-8 weeks old) were purchased from Beijing Vital River Laboratory Animal Technology Co., Ltd and housed in pathogen-free conditions with standard temperature and humidity conditions at Laboratory Animal Resources Center of Tsinghua University. All the animal experiments were approved by the Animal Ethics Committee of Tsinghua University (Approve no. 24-LJH3). 2 × 10^6^ MCF-7 cells were injected subcutaneously into the right flank of BALB/c nude mice to construct tumor-bearing mouse model.

# Supporting figures

^
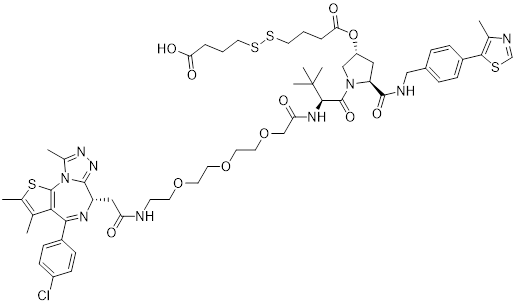

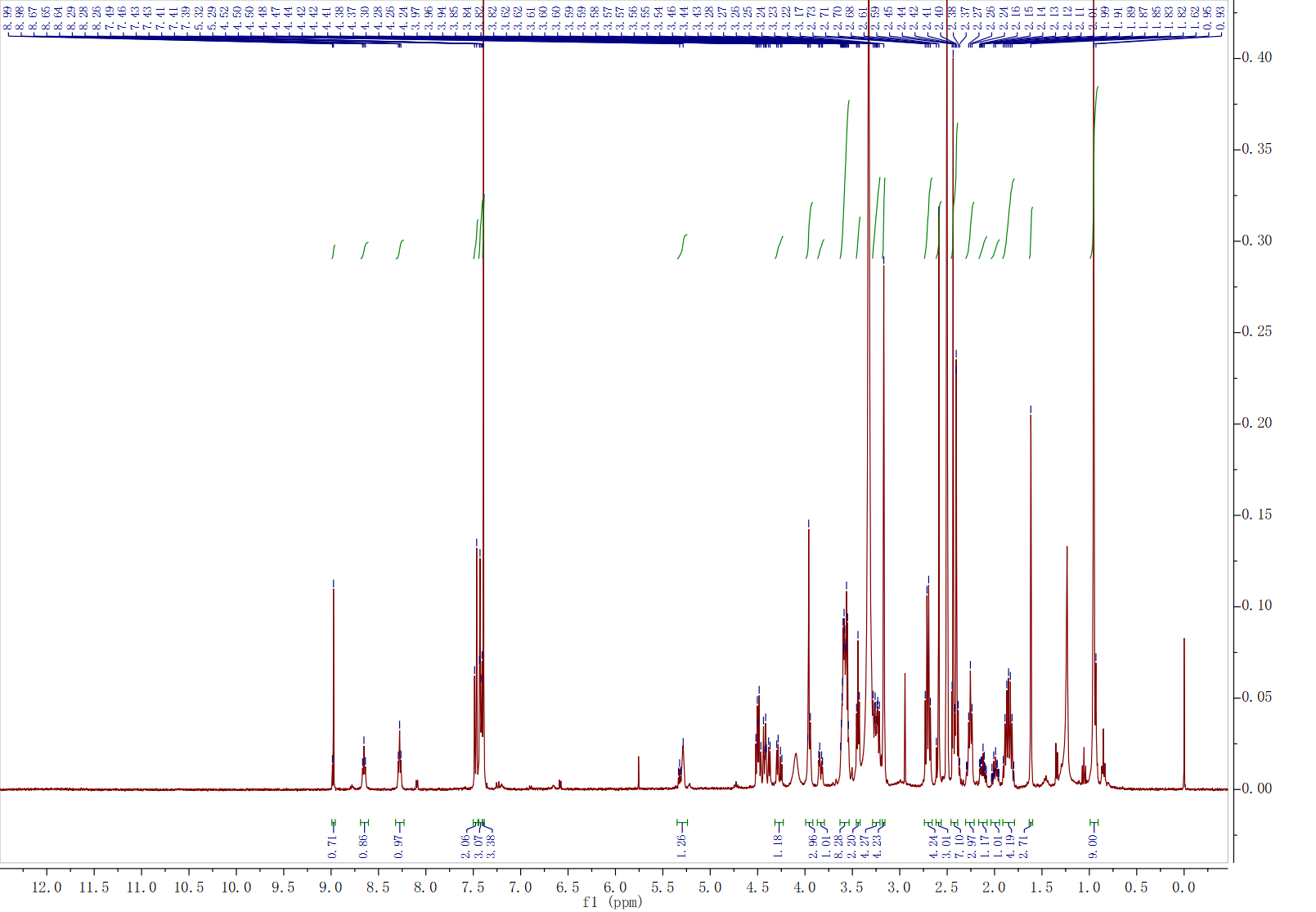
^

**Figure S1.** ^1^H NMR of MZ1-S-S-COOH.

**Figure S2.** HRMS (ESI) of MZ1-S-S-COOH.


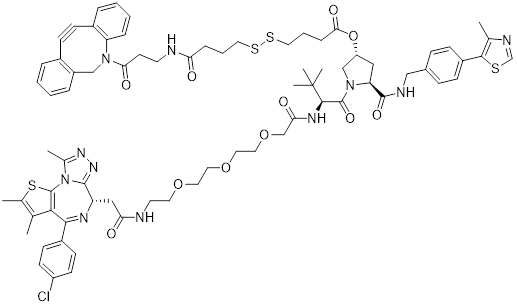

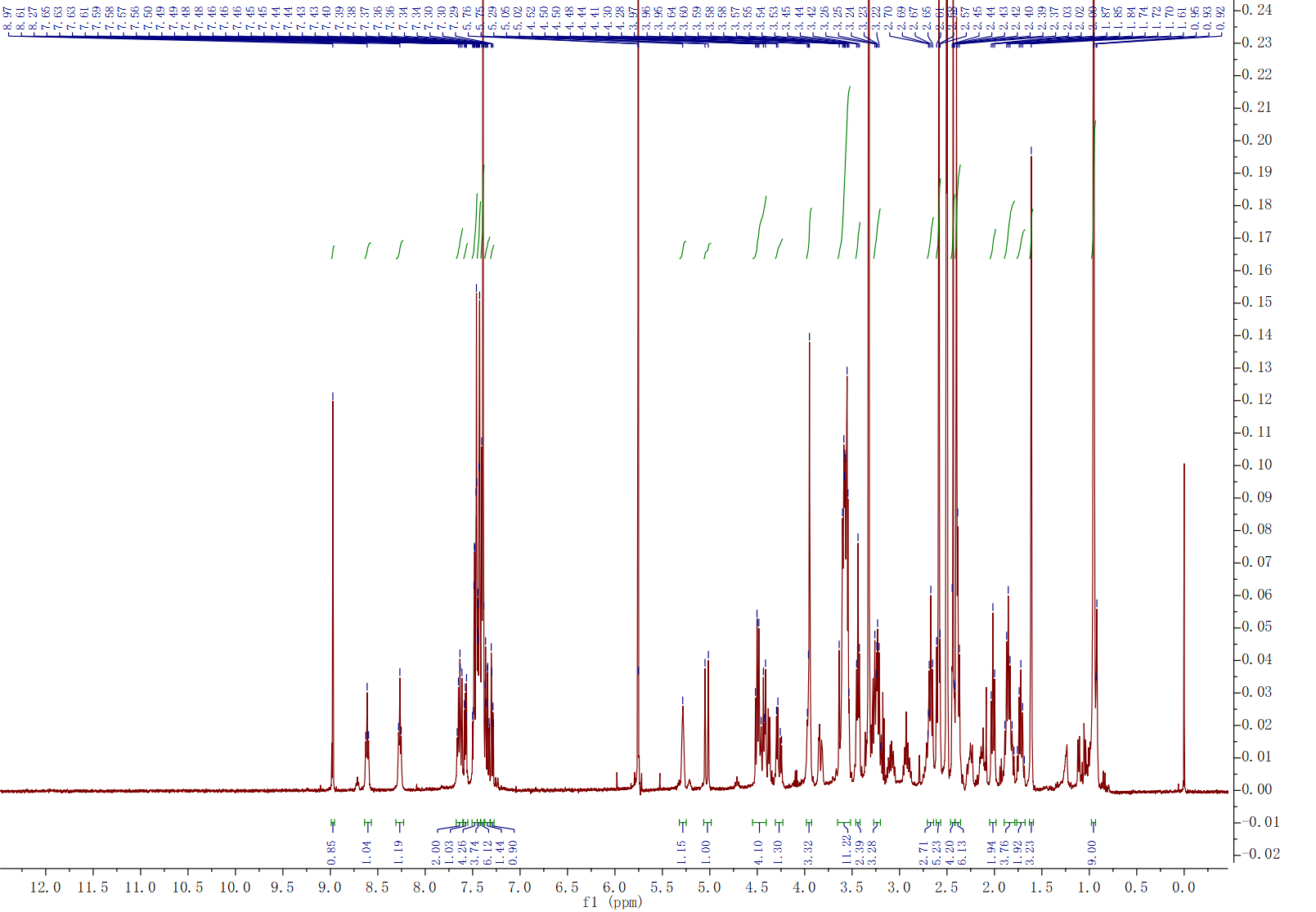


**Figure S3.** ^1^H NMR of MZ1-S-S-DBCO.

**Figure S4.** HRMS (ESI) of MZ1-S-S-DBCO.

**Figure S5****.** MALDI-TOF MS of PE-S-S-MZ1.


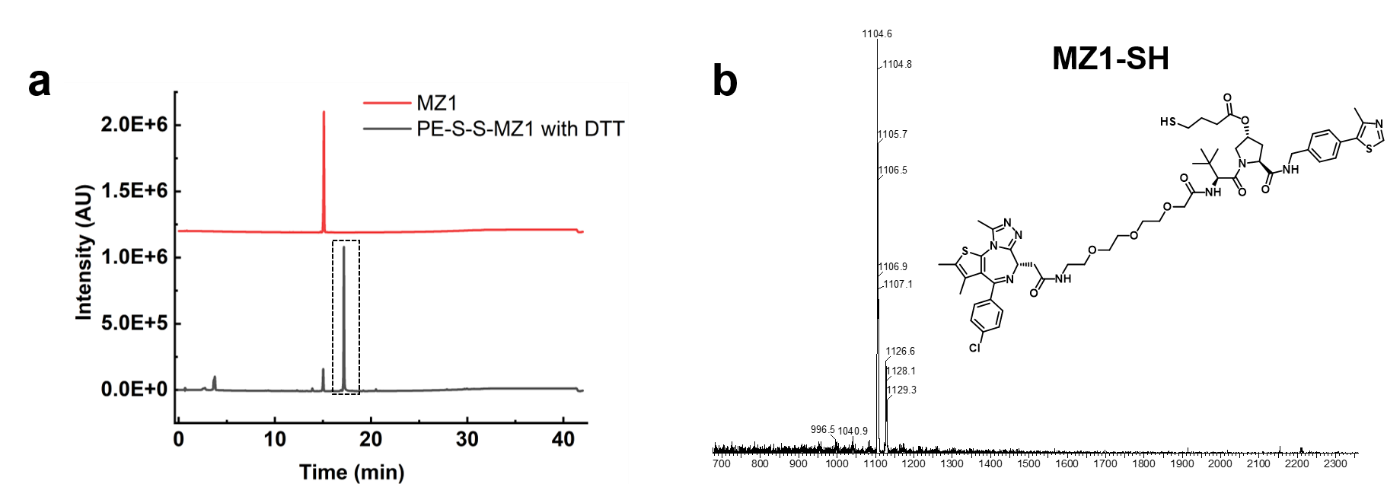


**Figure S6.** (a) The UPLC-mass spectrum of 100 μM MZ1 and 100 μM PE-S-S-MZ1 treated by 40 mM DTT for 2 h. (b) The MS of released MZ1-SH at R_T_ = 17.2 min of Figure S6 (a).


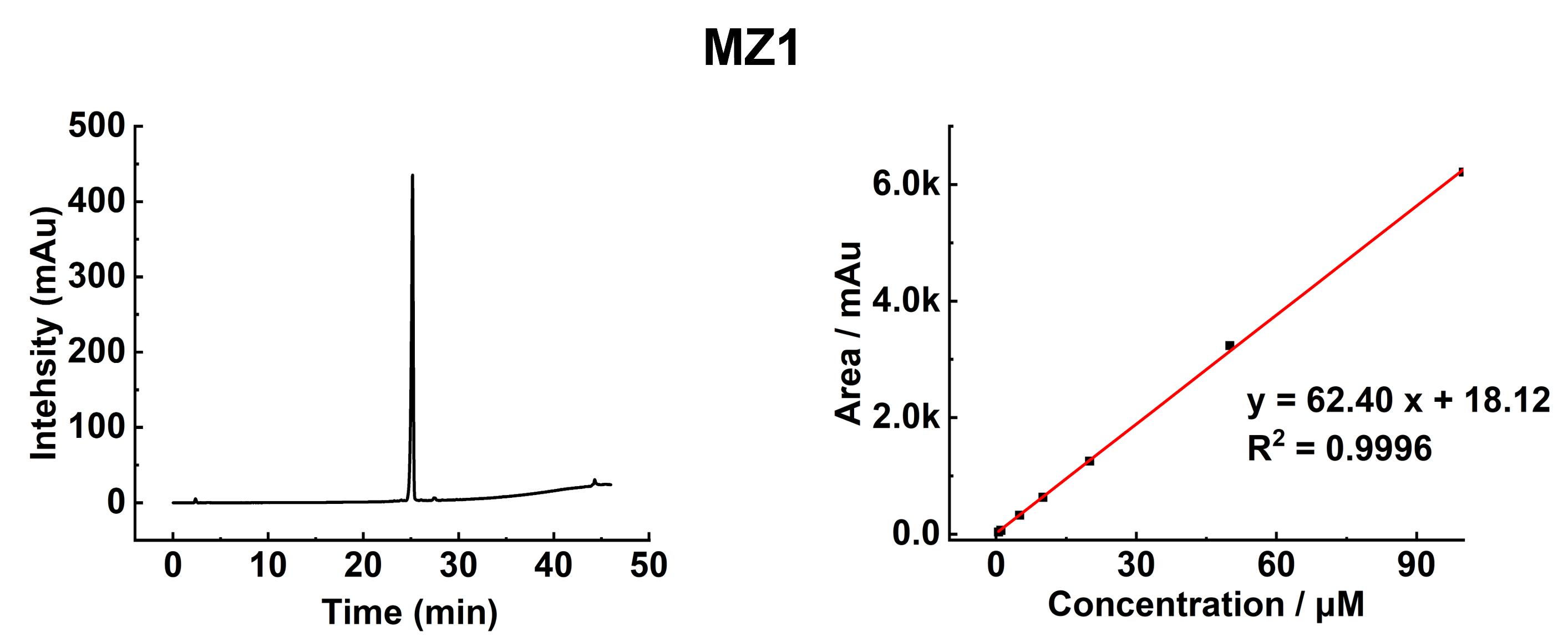


**Figure S7.** Respective representative HPLC chromatogram and standard curve for MZ1.

^
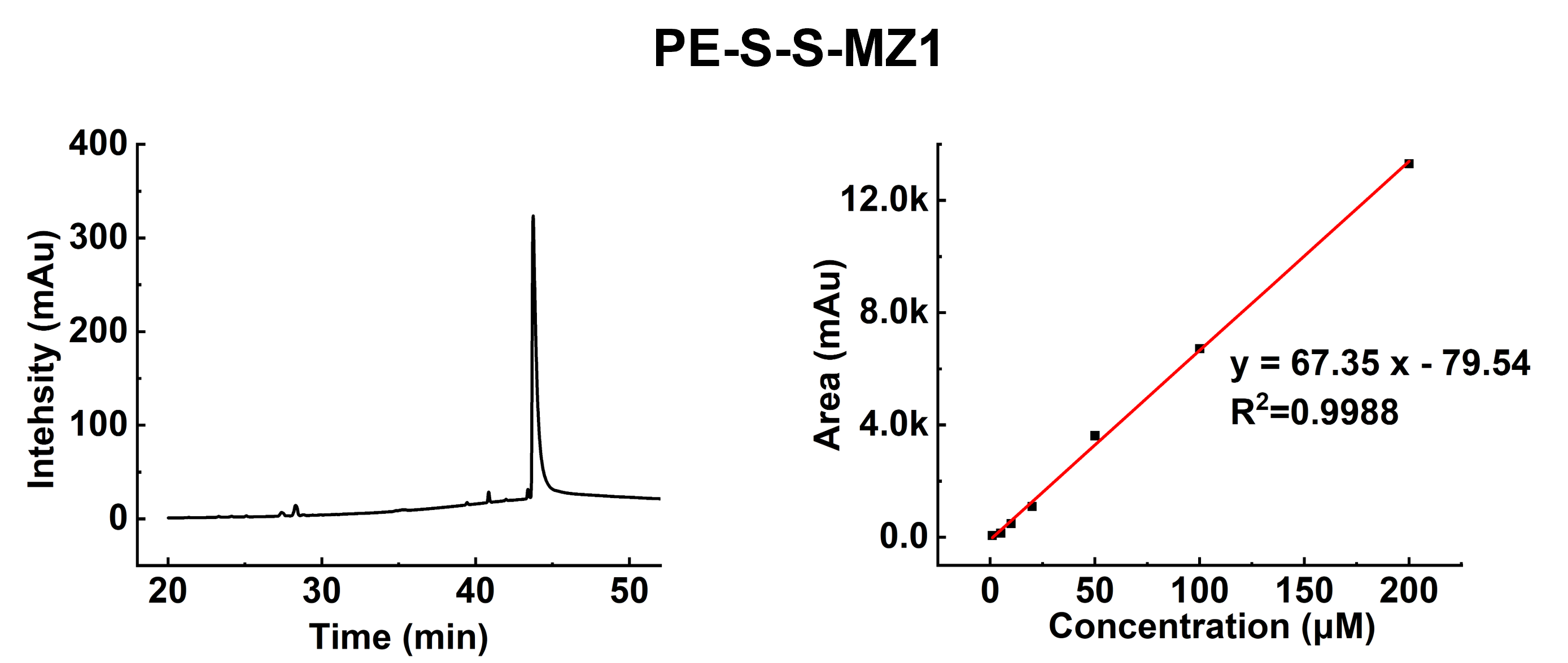
^

**Figure S8.** Respective representative HPLC chromatogram and standard curve for PE-S-S-MZ1.


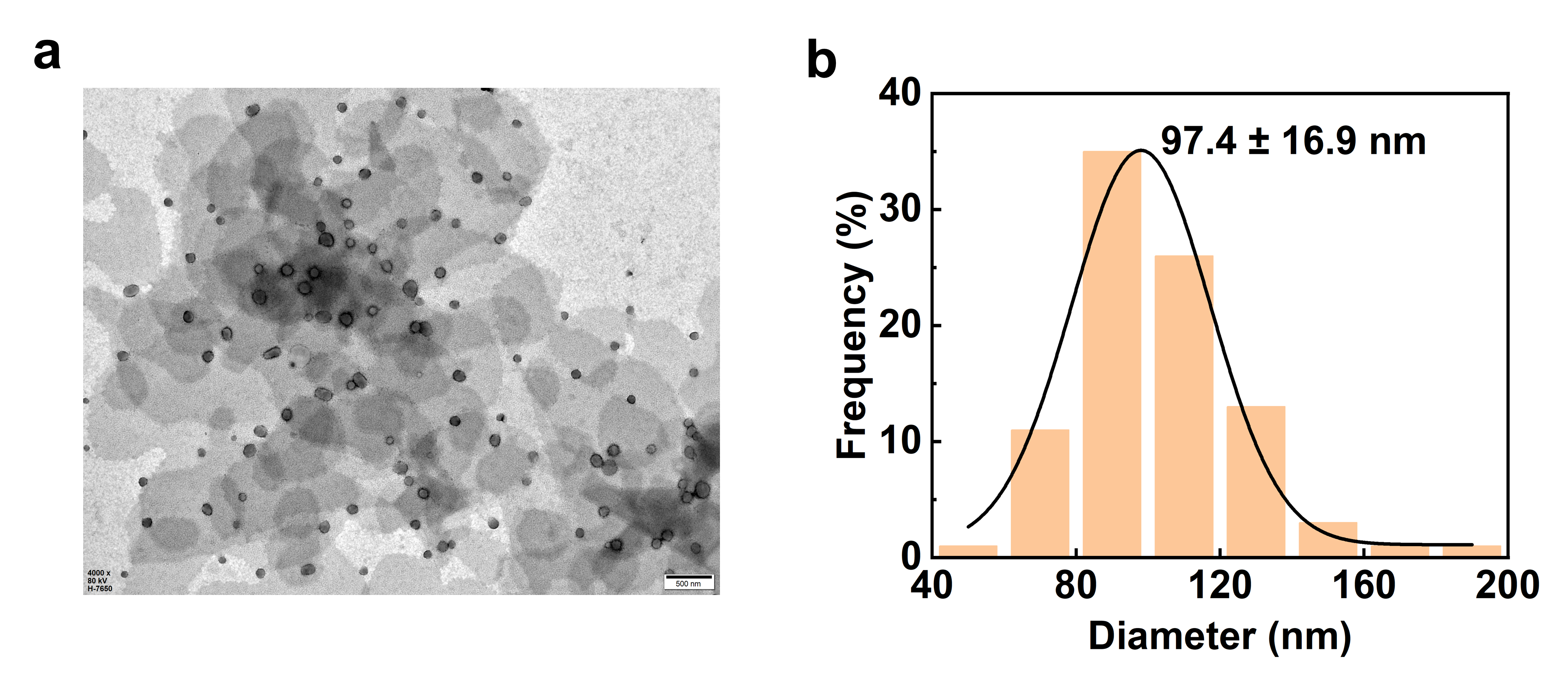


**Figure S9.** (a) Representative TEM image of the LND-MZ1 *via* 2% uranyl acetate negative staining. Scale bar = 500 nm. (b) Size distribution of LND-MZ1 derived from the TEM image of Figure S9a.


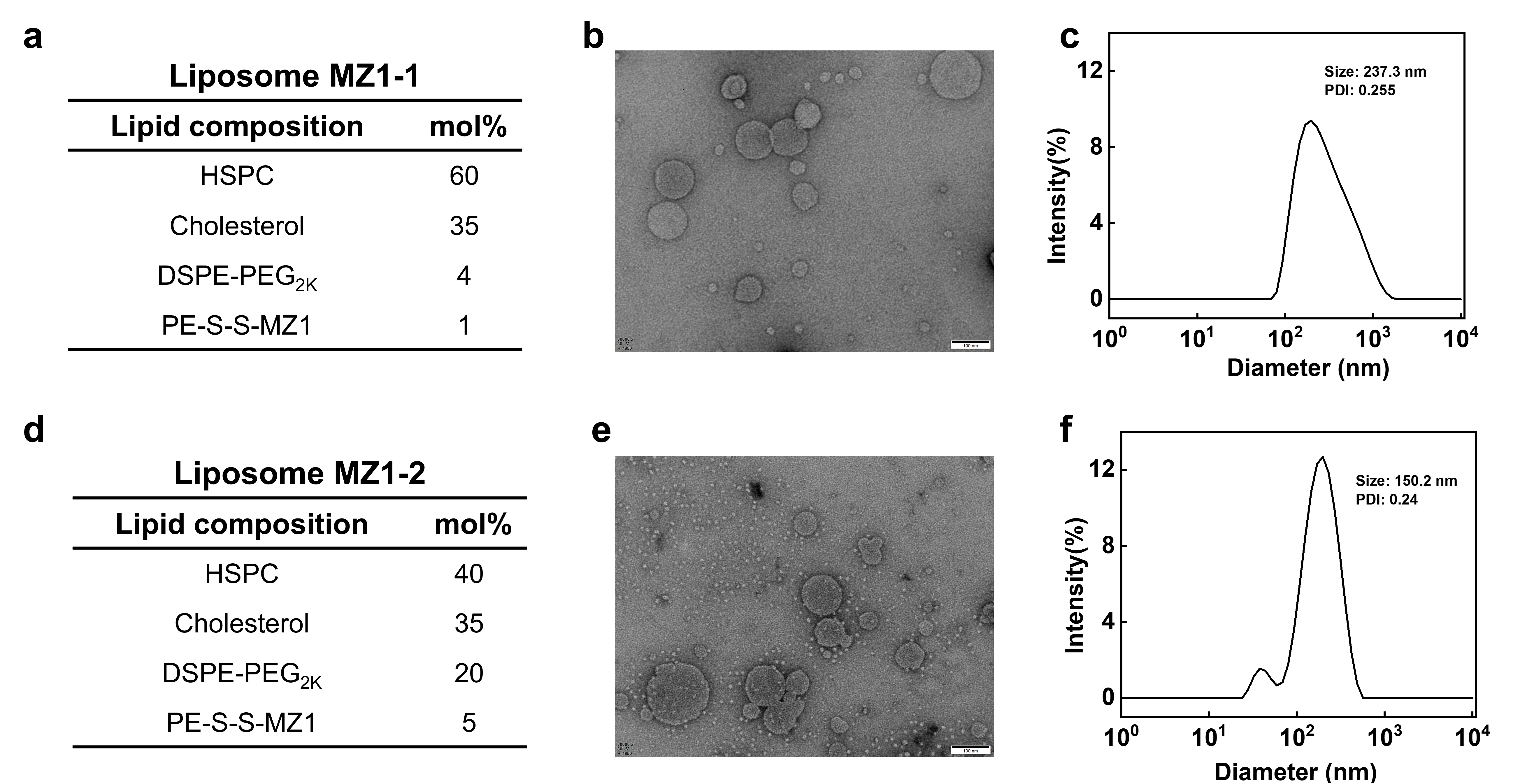


**Figure S10.** (a) Compositions, (b) a representative TEM image and (c) dynamic light scattering curve of liposome MZ1-1. (d) Compositions, (e) a representative TEM image and (f) dynamic light scattering curve of liposome MZ1-2. Representative TEM images were obtained using 2% uranyl acetate negative staining. Scale bar = 100 nm.

**
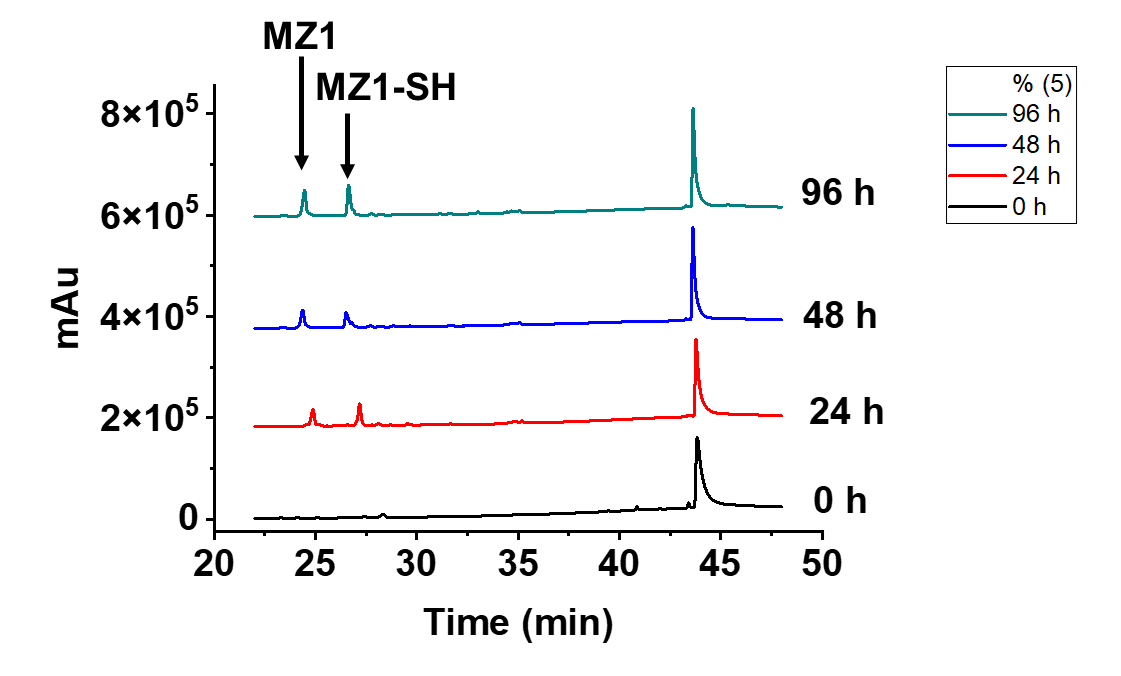
**

**Figure S11.** HPLC chromatograms of the released MZ1 from 100 μM LND-MZ1 by incubation with 10 mM GSH for different times at 37 ^o^C. The detection wavelength was 254 nm.

**Figure S12.** HRMS (ESI) of PE-Cy5.5.

**^
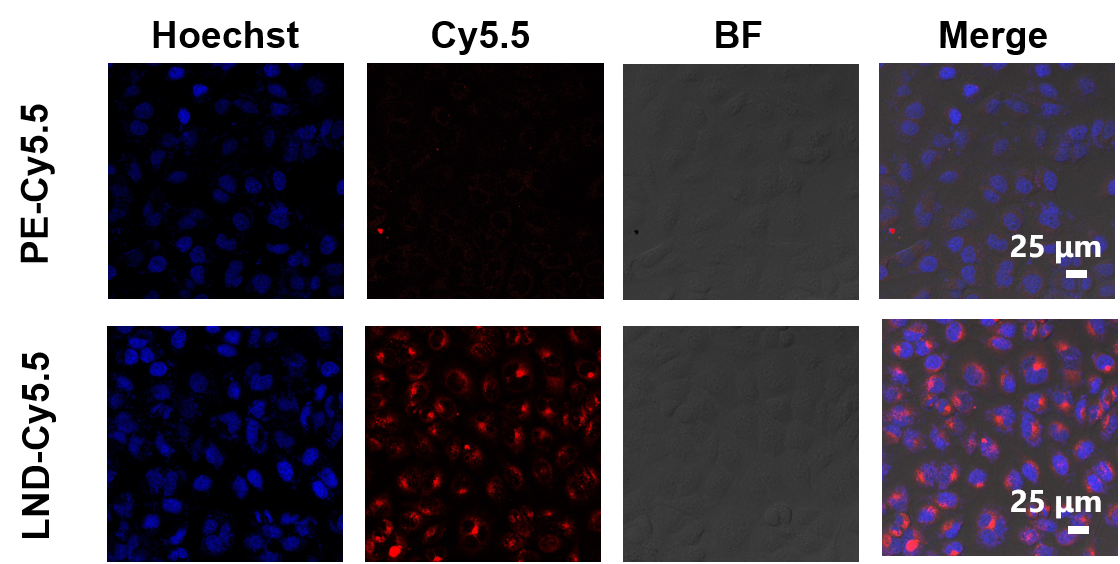
^**

**Figure S13.** CLSM images of intracellular distribution of the PE-Cy5.5 and LND-Cy 5.5 after incubation for 8 h followed by staining with Hoechst 33342 to evaluate cellular uptake performance (scale bar = 25 μm).


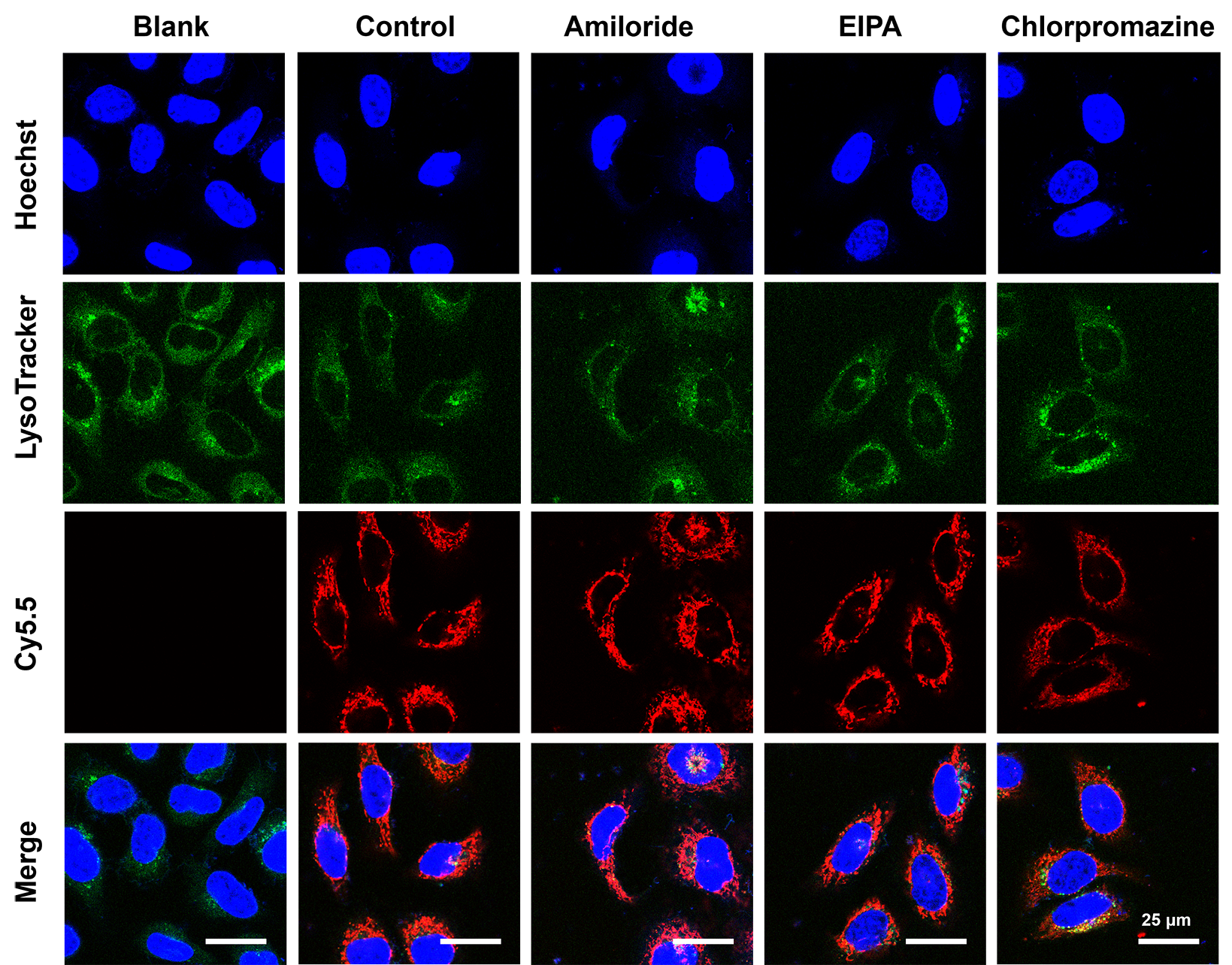


**Figure S14.** CLSM images of MCF-7 cells with LND-Cy5.5 and LysoTracker. After the treatment of different inhibitors for 30 min and the incubation of LND-Cy5.5 (5 μM) for 4 h, the cells were stained by Hoechst 33342 and LysoTracker for 15 min to locate the nuclei and lysosomes, respectively (scale bar = 25 μm).


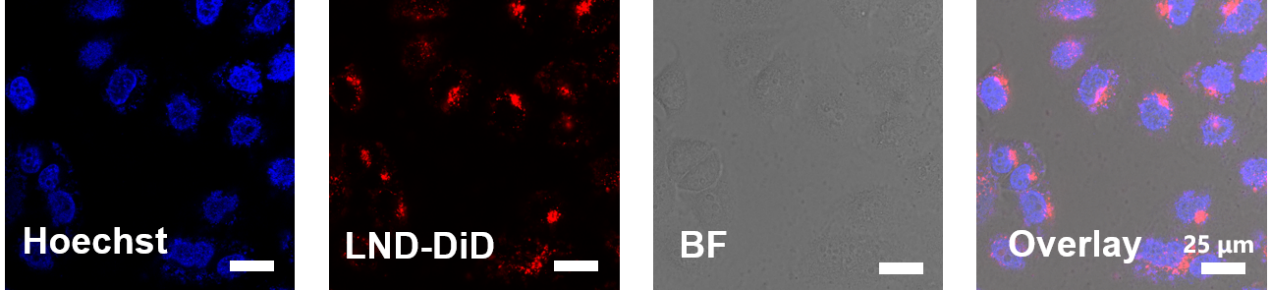


**Figure S15.** CLSM examination of intracellular distribution of the LND-Cy5.5 after 8 h of incubation followed by staining with Hoechst 33342 (scale bar = 25 μm).

**
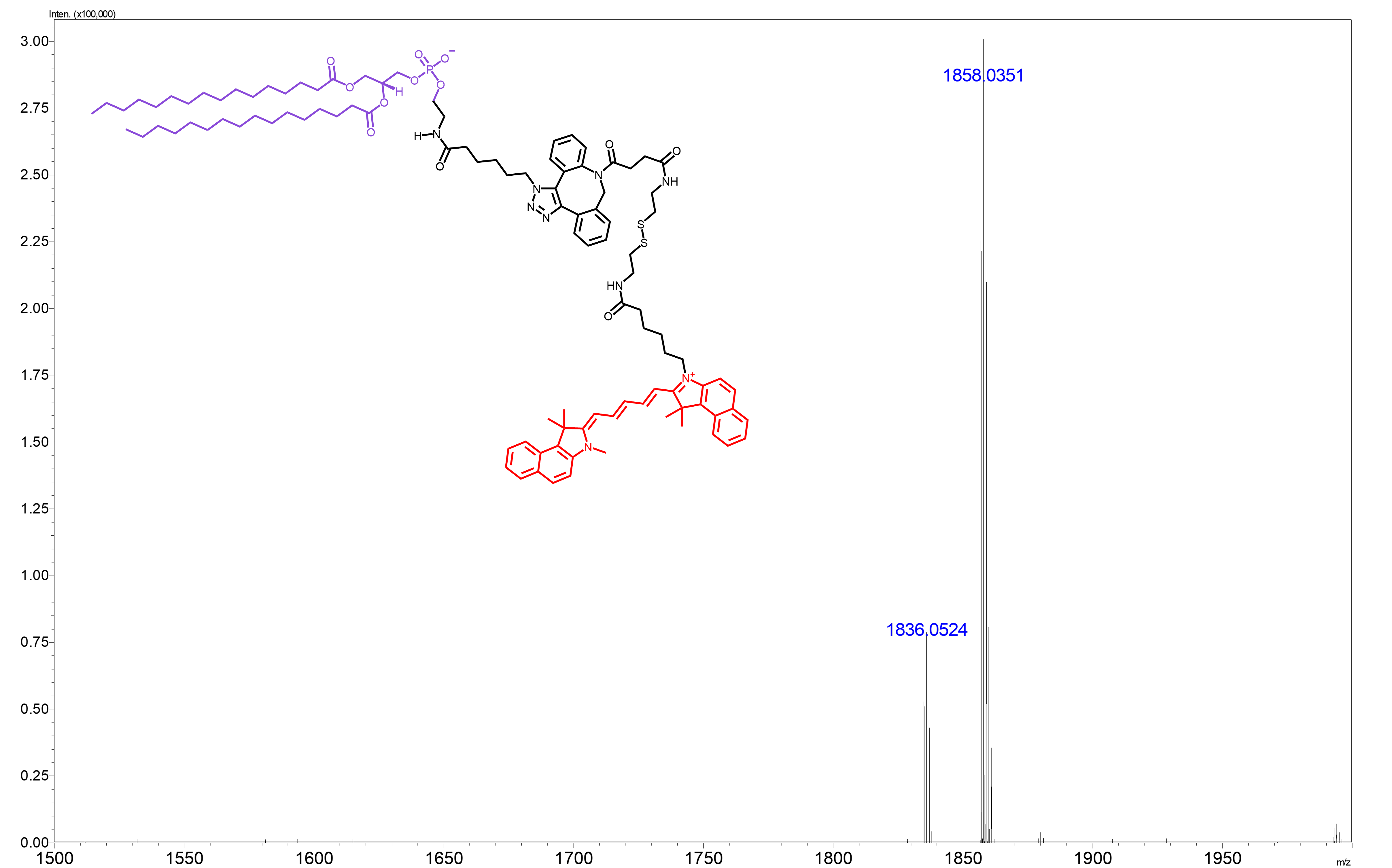
**

**Figure S16.** HRMS (ESI) of PE-S-S-Cy5.5.

**^
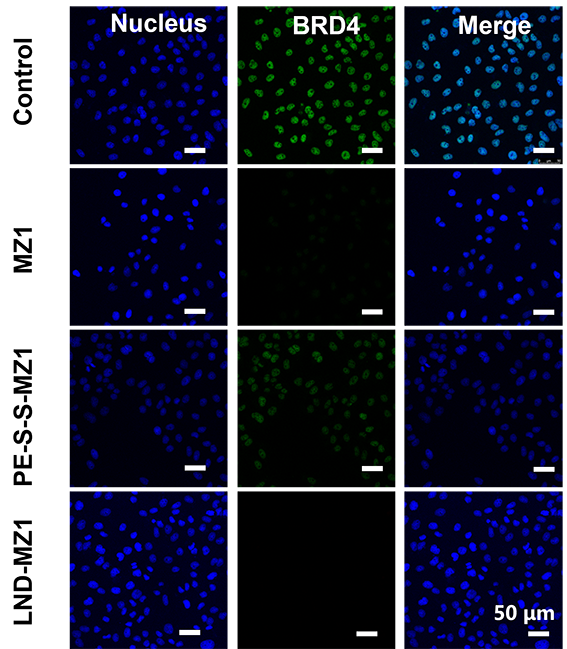
^**

**Figure S17.** Immunofluorescence staining images of MCF-7 cells which were untreated as control or treated by MZ1, PE-S-S-MZ1, LND-MZ1 with the concentration of 300 nM (scale bar = 50 μm).

**
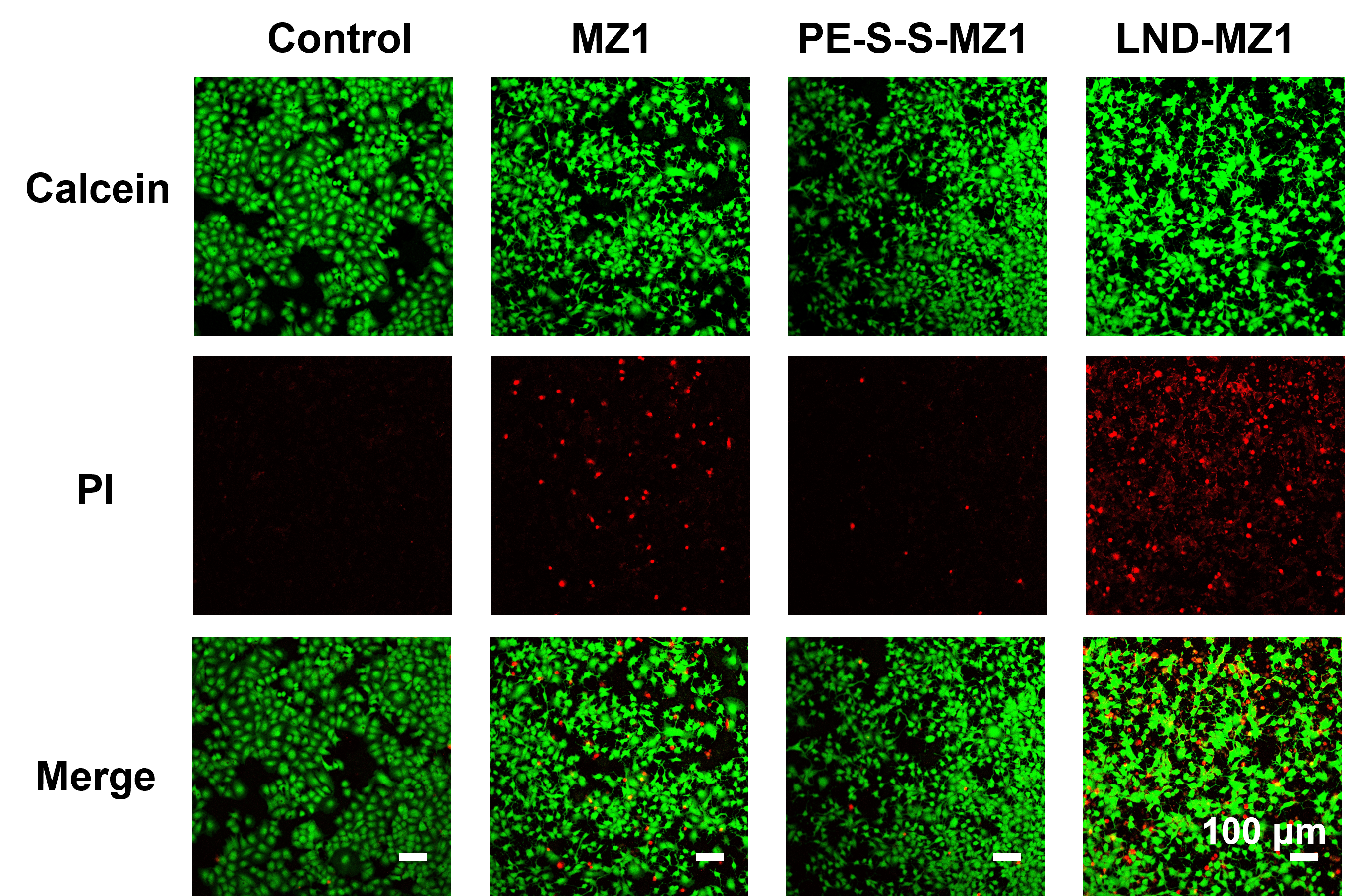
**

**Figure S18.** Calcein/PI cell viability of cells treated by MZ1, PE-S-S-MZ1, LND-MZ1 with the concentration of 300 nM (scale bar = 100 μm).


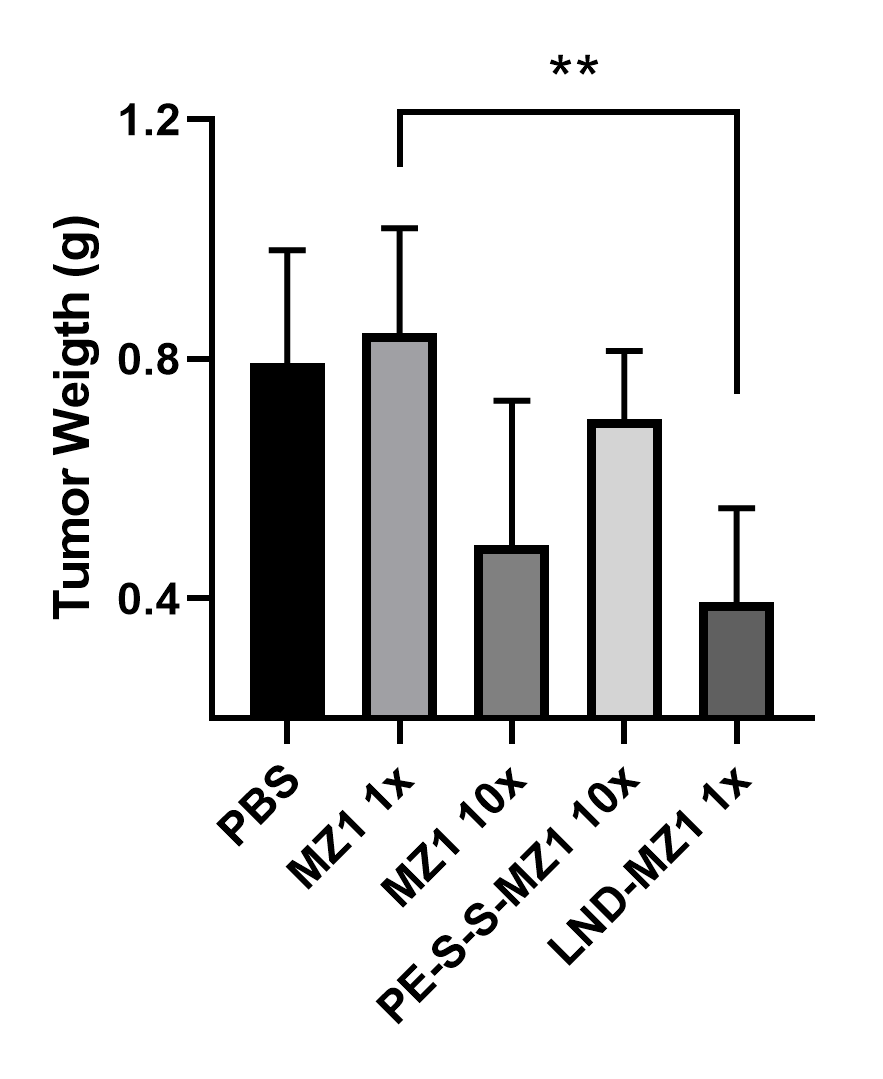


**Figure S19.** The tumor weights of the treatment groups, ***P* < 0.01.


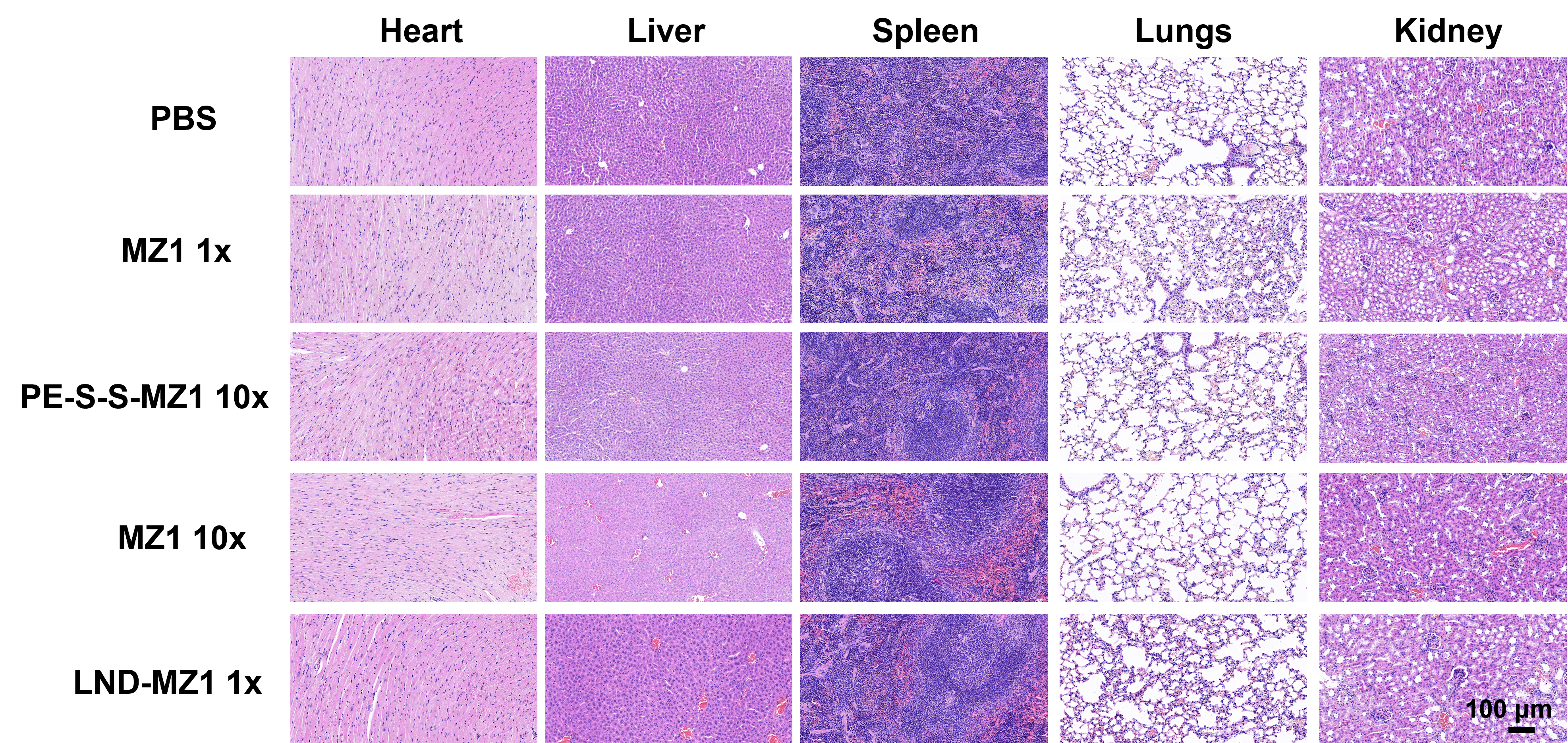


**Figure S20.** The H&E staining of tissues for histological analysis (scale bar = 100 μm) The tissues were harvested at day 20 after the treatment of PBS, MZ1 1× (2 mg/kg), MZ1 10× (20 mg/kg), PE-S-S-MZ1 10× (20 mg MZ1/kg) or LND-MZ1 1× (2 mg MZ1/kg).

**
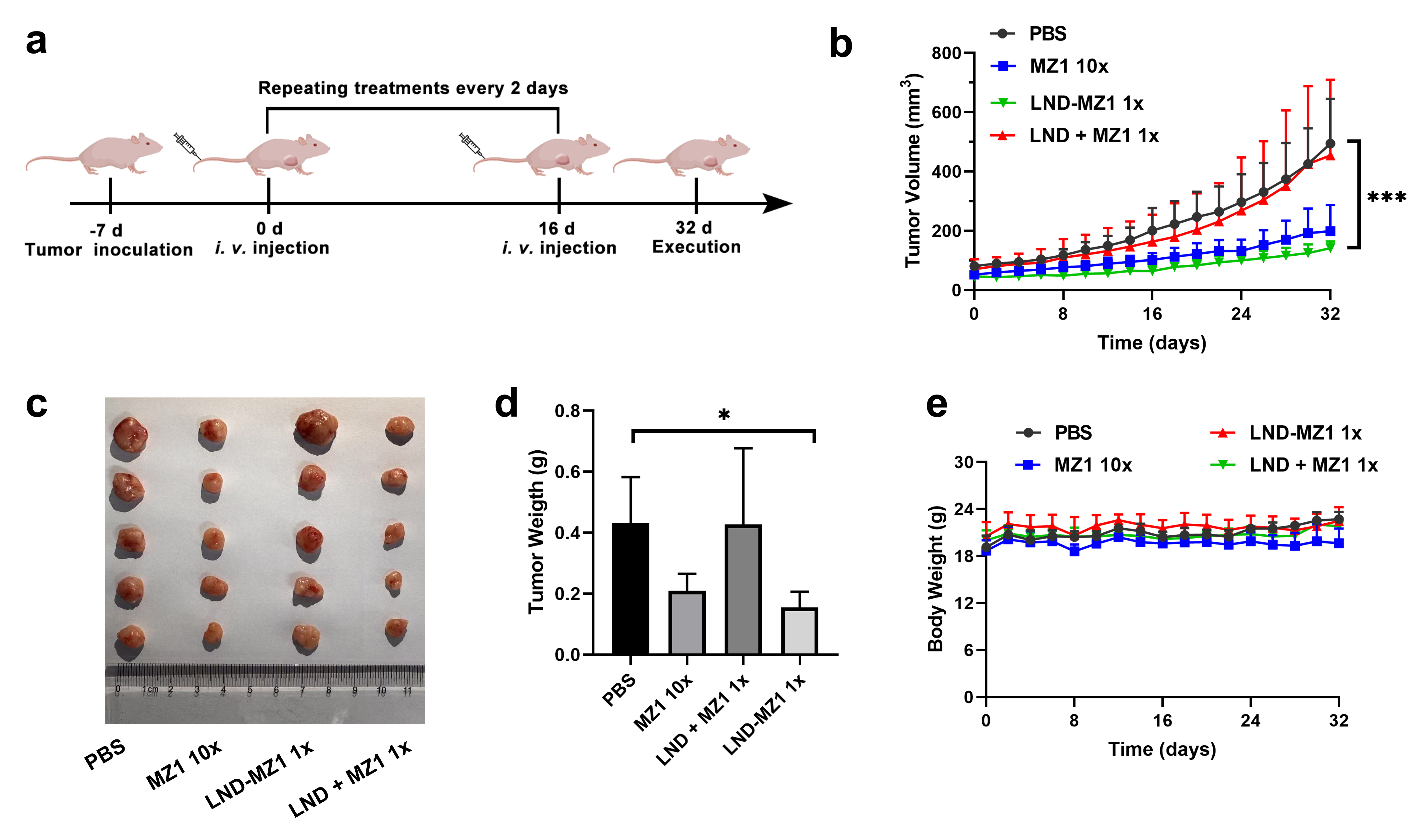
**

**Figure S21.** The assessment of antitumor efficacy and biosafety of LND-MZ1 in the MDA-MB-231 xenograft mouse model. (a) Schematic of the schedule for the antitumor study in tumor-bearing mice. From day 0, PBS, MZ1 10× (20 mg/kg), LND-MZ1 1× (2 mg MZ1/kg) or LND + MZ1 1× (2 mg MZ1/kg) were administered every 2-days via *i.v* injection for 16 days. (b) Tumor growth curves of the mice upon the treatment of different drugs during 32-day monitoring (n = 5 biologically independent mice), ****P* < 0.005. (c) Photographs of tumor tissues harvested at day 32. (d) Tumor weight of tumor tissues harvested at day 32, **P* < 0.05. (e) The mouse body weights of the treatment groups.


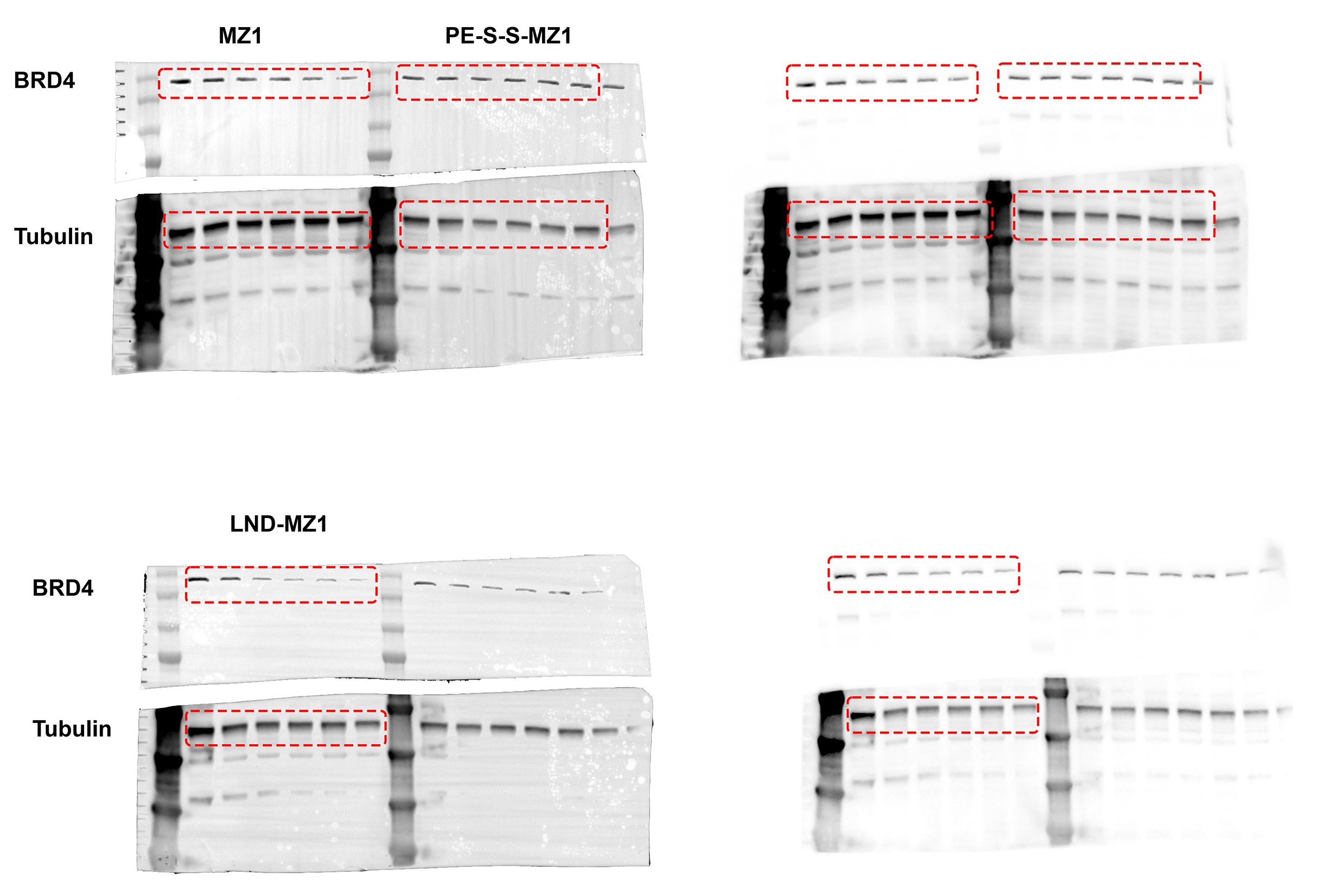


**Figure S22.** Uncropped western blotting source data for Figure 4a.


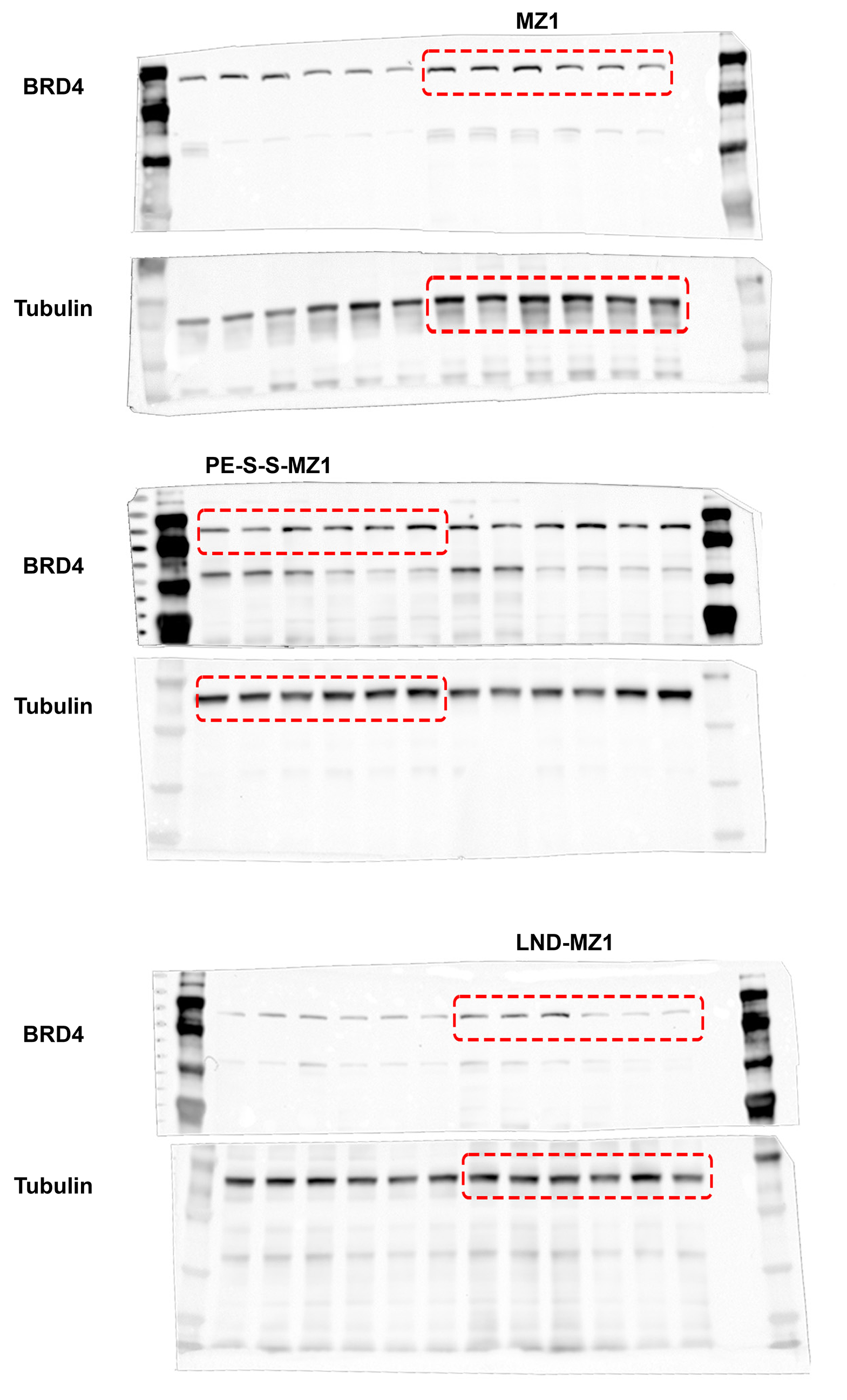


**Figure S23.** Uncropped western blotting source data for Figure 4b.


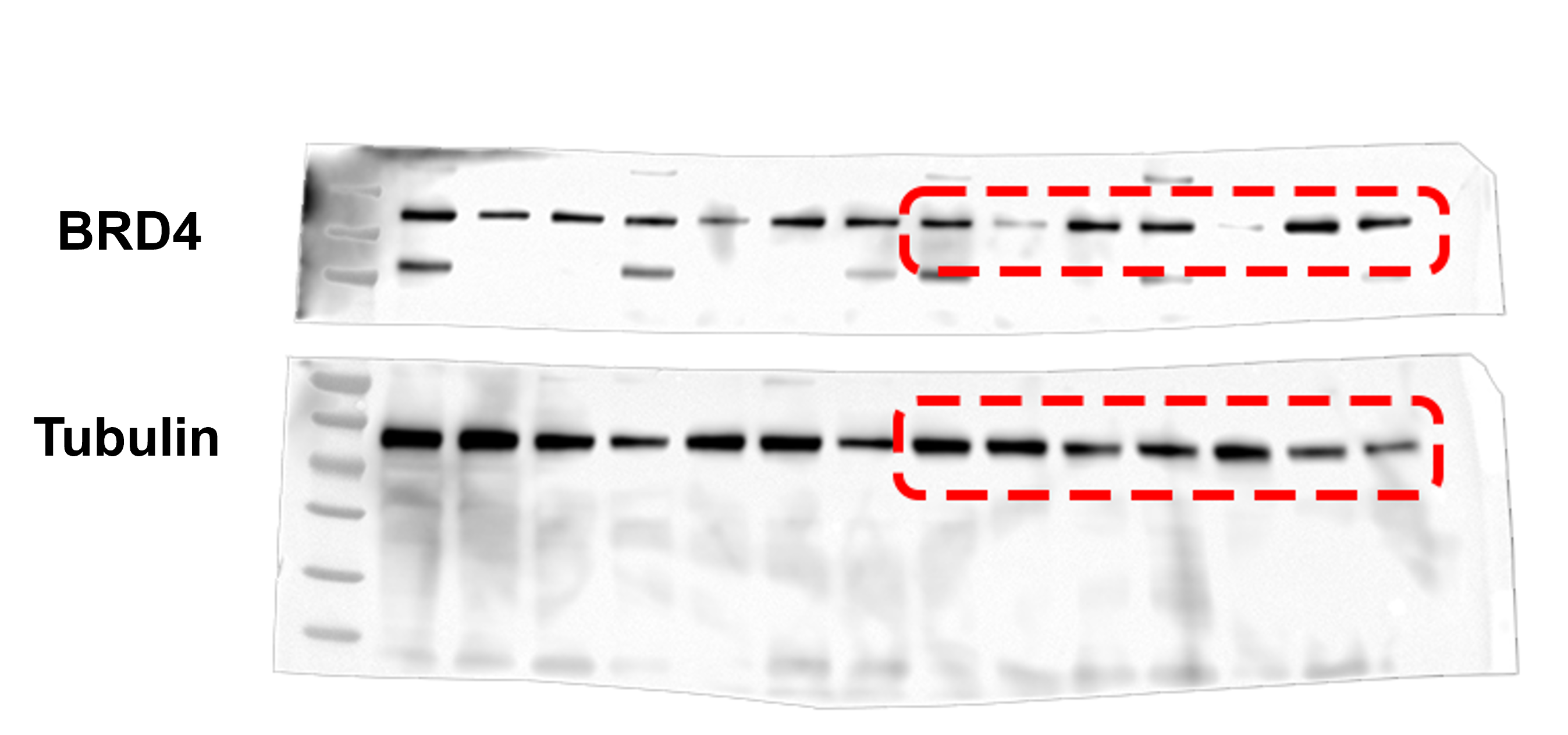


**Figure S24.** Uncropped western blotting source data for Figure 4c.
